# Attractive and healthy-looking male faces do not show higher immunoreactivity

**DOI:** 10.1038/s41598-022-22866-x

**Published:** 2022-11-01

**Authors:** Žaneta Pátková, Dagmar Schwambergová, Jitka Třebická Fialová, Vít Třebický, David Stella, Karel Kleisner, Jan Havlíček

**Affiliations:** 1 Faculty of Science, Charles University, Viničná 7, 128 44 Prague, Czech Republic; 2 Faculty of Physical Education and Sport, Charles University, José Martího 269, 162 52 Prague, Czech Republic; 3 Global Change Research Institute of the Czech Academy of Sciences, Bělidla 986/4a, 603 00 Brno, Czech Republic

**Keywords:** Human behaviour, Sexual selection

## Abstract

Previous research has indicated that facial attractiveness may provide cues to the functioning of the immune system. Mating with individuals who have a more effective immune system could lead to a higher reproductive success. Our main aim was to test a possible association between immunoreactivity (stimulated by vaccination) and perceived facial attractiveness and healthiness. We experimentally activated the immune system of healthy men using vaccination against hepatitis A/B and meningococcus and measured levels of specific antibodies (markers of immune system reactivity) before and 30 days after the vaccination. Further, 1 day before the vaccination, we collected their facial photographs that were judged by females for attractiveness, healthiness, and facial skin patches for healthiness. In view of its proposed connection with the functioning of the immune system, we also measured skin colouration (both from the facial photographs and in vivo using a spectrophotometer) and we assessed its role in attractiveness and healthiness judgements. Moreover, we measured the levels of steroid hormones (testosterone and cortisol) and the percentage of adipose tissue, because both are known to have immunomodulatory properties and are related to perceived facial attractiveness and healthiness. We found no significant associations between antibody levels induced by vaccination and perceived facial attractiveness, facial healthiness, or skin healthiness. We also found no significant connections between steroid hormone levels, the amount of adipose tissue, rated characteristics, and antibody levels, except for a small negative effect of cortisol levels on perceived facial healthiness. Higher forehead redness was perceived as less attractive and less healthy and higher cheek patch redness was perceived as less healthy, but no significant association was found between antibody levels and facial colouration. Overall, our results suggest that perceived facial attractiveness, healthiness, and skin patch healthiness provide limited cues to immunoreactivity, and perceived characteristics seem to be related only to cortisol levels and facial colouration.

## Introduction

Mate preferences are often based on physical appearance, whereby facial attractiveness seems to play an especially significant role^1^. It is often claimed that facial attractiveness provides cues to various aspects of individuals’ quality, such as immunocompetence^1–3^. Selection of partners with a more effective immune system is expected to lead to a higher reproductive success by passing increased pathogen resistance onto the offspring (indirect benefits). Moreover, healthier individuals can provide better parental care and are less likely to transmit any infections to their partners (direct benefits)^1,4^.

Previous research into the putative relationship between facial attractiveness and individual's quality that was conducted using self-reported past and current health and attractiveness ratings of facial photographs delivered mixed results^5,6^. Several recent studies employed direct immunity function measures, such as inflammation markers or levels of cytokines or antibodies. In a sample of South African men, Phalane et al.^7^ tested the relationship between facial attractiveness ratings and responsiveness of the immune system upon activation by an injection of bacterial lipopolysaccharide (LPS). Immune system response was assessed by levels of C-reactive protein, which is an inflammation marker, and by the levels of cytokines, which are peptides that stimulate the immune response. This study found a positive correlation between facial attractiveness ratings and the levels of cytokines, specifically interleukins (IL)-2, 4, 6, 8, and 10, granulocyte–macrophage colony-stimulating factor (GM-CSF), interferon γ (IFN-γ), and tumour necrosis factor α (TFN-α)^7^. Other studies employed vaccinations to elicit and measure immune system reactivity. A stronger response to the vaccine (assessed via higher antibody levels) indicates a better protection against infection^8^. Overall, though, the results of these studies are inconclusive. Faces of men with higher levels of hepatitis B antibodies were rated as more attractive^9^ but this did not hold of women^10^. In contrast, other study reported a negative, though nonsignificant, association between facial attractiveness and immune system reactivity in men^11^.

It has been suggested that facial skin colouration plays an important role in perceived facial attractiveness and health^12–15^. Studies tend to focus on facial skin colouration in the CIE L*a*b* colour space^16,17^. Higher skin redness (a*) is linked to increased skin blood perfusion and oxygenation^16^, which are in turn positively associated with physical fitness^18,19^ as well as good cardiovascular^20^ and pulmonary health^16,18^. Skin yellowness (b*) is influenced by carotenoids, which are pigments acquired from food, mainly fruits and vegetables. Owing to their antioxidant properties^21^, carotenoids can contribute to disease resistance as they can destroy free radicals and reduce oxidative stress, both of which are harmful to the immune system^22,23^. It has been shown that facial skin with higher redness and yellowness is perceived as more attractive and healthier^17,24^. Moreover, Phalane et al.^7^ reported an association between skin yellowness and a marginally higher immune system response (higher levels of inspected cytokines) after LPS stimulation. On the other hand, Foo et al.^25^ found that higher skin yellowness is positively associated only with perceived health (and only in men) and not with direct immune function measures^25^. Skin lightness (L*) is determined by the distribution pattern of melanosomes in keratinocytes and the amount of melanin it contains^26^. Higher melanin levels (resulting in a darker skin hue) can provide a better protection against sunlight^27^ but can also contribute to vitamin D deficiency^28^. It has been found that melatonin can have an effect on the synthesis of melanin^29^, which is in turn believed to affect the periodicity of immune response as well as cytokine production^30,31^. In women, lighter skin is associated with higher perceived attractiveness and youth^32–34^ (but see Fiala et al.^35^), because with increasing age skin tends to become darker^36^. In men, some research shows that darker complexion may be preferred^37^.

The association between functioning of the immune system and perceived facial attractiveness might be also modulated by testosterone and cortisol. It has been suggested that testosterone has an immunosuppressive effect^38–40^ but evidence to that effect is rather mixed^41^. It has thus been proposed that glucocorticoids, such as cortisol, mediate the association between testosterone and the immune system functioning^42,43^. Although a short-term elevation of cortisol levels can boost an acute immune system response, prolonged exposure may weaken the response, thereby increasing susceptibility to diseases^44^. Some support for the mediating effect of cortisol comes from Rantala et al.^9^ who found that immunoreactivity was stronger in men with higher testosterone and simultaneously lower cortisol levels, while immunoreactivity was also positively linked to facial attractiveness. Similarly, women with lower cortisol levels were perceived as more attractive^10,45^ (for null results see Han et al.^46^).

Another key factor affecting both attractiveness and immunity is adiposity. Obesity contributes to an altered immune function and reduced immunocompetence because it is associated with changes in leucocyte counts, reduced antibody production, impaired wound healing, a higher risk of infections, and even a higher mortality rate^47–50^. In perception studies, body fat levels affect attractiveness ratings, whereby both overweight and excessively thin individuals are perceived as less attractive^5,10,51^. Moreover, portrait photographs of individuals with elevated levels of leptin—a hormone produced by the adipose tissue that has a negative effect on health—were also perceived as less attractive^52^.

Overall, evidence pertaining to links between the quality of the immune system and facial attractiveness is ambiguous. Many previous studies investigated only a limited number of relationships between variables and relied on indirect measures of immune system functioning. In Study 1, we therefore focused on the relationship between immune system reactivity and perceived facial attractiveness. To measure the reactivity of the immune system, we experimentally activated the immune system by vaccination against both viral (hepatitis A, B) and bacterial (meningococcus) infections, because the two in conjunction should stimulate a wider range of components of the immune system than either would. We used differences in antibody levels before and after vaccination as a proxy for reactivity of the immune system. In Study 2, we investigated associations between immune system reactivity and perceived skin patch healthiness to examine human ability to judge characteristics from limited amount of information. Finally, in Study 3 we focused on the relationship between immune system reactivity and perceived healthiness of the face. Moreover, we measured testosterone and cortisol levels and recorded body composition, because all these factors have immunomodulatory properties and are linked to both perceived facial attractiveness and healthiness. We also measured facial skin colouration (both from the facial photographs and in vivo using a spectrophotometer) to assess its role in attractiveness and healthiness judgements and its connection to the immunoreactivity.

## Materials and methods

Data used for this study are part of a larger project which investigates possible associations between reactivity of the immune system and attractiveness of human body odour^53^, face, and voice as perceived by opposite-sex individuals. The present article focuses on associations between immune system reactivity and perceived facial attractiveness, healthiness, and skin healthiness. All procedures were conducted in accordance with the Helsinki Declaration and the study was approved by the Institutional Review Board of Charles University (approval no. 20/2016). Due to the nature of this study, we have collaborated with medical personnel. The study was preregistered prior to data analyses (https://osf.io/69zgc). Before entering the study, all participants were informed about its goals and expressed their consent with participation by signing an informed consent form.

### Targets

We have collected data from 21 men (mean age = 26.2 ys, SD = 4.62, age range = 20–35 ys). Requirements for participating in the study were the following: age 18–40 years, good general health, no current use of any medication, non-smokers, and not being vaccinated against hepatitis A, B, or the meningococcus in the past 10 years (e.g., Shepard et al.^54^).

Participants were recruited via social media advertisements (Facebook) and leaflets at university halls of the Faculty of Science, Faculty of Humanities, and Faculty of Physical Education and Sports (all of the Charles University, Prague, Czechia). Participants were vaccinated free of charge and received a reimbursement of 400 CZK (approx. €15) for participation in the whole project as a compensation for their time and potential inconvenience. Targets were the same for all studies described in the present article (Studies 1–3).

### Procedure

One day before vaccination, we acquired standardised portrait photographs of the participants. On the day of the vaccination, each participant completed a questionnaire on their medical history and their general health status was examined by a physician to ensure they were eligible for application of the vaccines and not suffering from any current illness or infection. This was followed by the first blood collection (5 ml of venous blood) to assess the basal levels of antibodies (specific immunoglobulins G—IgG and immunoglobulins M—IgM) and C-reactive protein (CRP), which is a marker of inflammation. In none of the participants did the pre-vaccination CRP levels exceed 5.5 mg/l; values below this threshold are considered clinically normal^55^, that is, such values do not indicate currently ongoing infection. After the blood collection, the vaccines against hepatitis A/B (Twinrix) and meningococcus (Menveo) were administered. We selected vaccines against both viral and bacterial infections to stimulate different components of the immune system (nonspecific, specific, cellular, and humoral). The second blood collection and second photograph acquisition took place 14 days after vaccination, at a time point when one should expect the highest antibody response^56^. For the current investigation, only photographs taken before the vaccination were used. The last blood collection took place 30 days after vaccination, at a point when a second dose of vaccine against hepatitis (Twinrix) is recommended^57^. Vaccination and first blood collection were performed by a physician, while the remaining two blood samples were collected by phlebotomists at the Prevedig laboratory (https://www.prevedig.cz/) where all samples were subsequently analysed. The procedure and time of blood collections were standardised across participants. To avoid diurnal fluctuations^58^ sampling was conducted at 7–8 a.m. We measured and recorded body composition of the participants. Participants also completed questionnaires about their health status during the study and about possible factors that may have influenced their skin colour (e.g., traveling abroad to a sunny destination, use of tanning beds, self-tanning creams, or the consumptions of vegetables and fruits with high levels of carotenoids)^59,60^. This procedure took place on Q4 2017 to minimise possible effects of a suntan.

### Vaccine characteristics

To induce an immune system response, we used the Twinrix Adult vaccine against hepatitis A/B and a Menveo vaccine against meningococcus (which prevents meningococcal diseases caused by *Neisseria meningitis* serogroups A, C, Y, and W-135). They can be administered together and are widely used in the Czech Republic. Both were applied intramuscularly, each in one arm.

### Laboratory assays

All laboratory analyses worked with the serum or plasma and were performed in a certified Prevedig laboratory. Total level of antibodies against hepatitis A (Anti-HAV) were measured by the Diasorin® Liaison—chemiluminescence immunoassay (CLIA), where a fully automated immunological analyser performs the full processing of samples. We used the corresponding Human S100 CLIA kits. This analysis is based on a radioimmunoassay, where the antigen and paramagnetic microparticle solid phase binds with fluorescent-labelled antibodies and after oxidation–reduction reaction, excessive energy is released in the form of photons^61^. The final photometric measurement and evaluation were done by the analyser.

Antibodies against hepatitis B (Anti-Hbs) were measured based on the same principle as Anti-HAV. It turned out, however, that large percentage of targets either had high levels of Anti-Hbs at the baseline (N = 7) or did not respond to vaccination (N = 5). For this reason, the Anti-Hbs were excluded from further analyses.

Antibodies against the meningococcus (Anti-Mnk) were measured by the fully automated Diasorin® ETI-Max 3000—enzyme immunoassay (ELISA), one of the basic methods of determination of serum antibodies. The method is based on a reaction between an antigen on a special board and antibodies in the patient’s serum. Then secondary antibodies are added, which are specially labelled and bind to the primary antibodies with the antigen. A chromogenic substrate, which is added last, causes a colour response that is measured by spectrophotometer^62^. Sufficient response is at least 1:4 titres (the dilution of the serum where antibodies still react with the antigens) and ideally even higher^63^. As above, the final photometric measurement and evaluation were conducted by the analyser.

Total testosterone levels were measured by chemiluminescence (CLIA) in a fully automatised analyser Beckman Coulter DxI 800 Immunoassay System. The CLIA principle is described above. In this case, the energy is released by a reaction between testosterone, polyclonal anti-testosterone antibodies, and a tracer^64^. The final photometric measurement and evaluation were done by the automatised analyser.

Cortisol levels were measured by an electrochemiluminescence immunoassay method in a fully automatised analyser Beckman Coulter DxI 800 Immunoassay System. First, one incubates a sample in which specific anti-cortisol antibodies labelled with ruthenium chelate bind to cortisol. This complex is captured on the surface of an electrode where the electric charge causes a chemiluminescent emission of photons. The emitted light is measured by a spectrophotometer, but the measurement and evaluation are likewise done by the analyser.

### The acquisition of photographs

Acquisition of photographs took place at the Human Ethology perception lab in a purpose-built photographic booth in order to prevent potential changes in ambient illumination and colour reflections^65^.

Portrait photographs were taken with a 24-megapixel full-frame (35.9 × 24 mm CMOS sensor, a 35 mm film equivalent) digital SLR camera Nikon D610 equipped with a 85 mm fixed focal length lens^66^ (Nikon AF-S NIKKOR 85 mm f/1.8G) into 14-bit uncompressed raw files (.NEF) and Adobe RGB colour space. The camera was mounted in a portrait orientation directly on a light stand that also carried a strobe light. A single 400Ws studio strobe (Menik MD-400Ws) was used and equipped with a white reflective umbrella light diffuser (Photon Europe, 109 cm diameter) mounted onto a 175 cm high light stand tilted 10° downwards toward the booth. Correct and uniform exposure across the entire scene was checked before each session with a digital light meter (Sekonic L-308S). Colour calibration was performed using X-Rite Color Checker Passport colour targets and a white balance patch photographed at the beginning of each session. For further details of the photo acquisition procedure, see Třebický et al.^65^.

Participants were photographed wearing provided white T-shirts and without any adornments or glasses. They had varying amount of facial hair ranging from clean-shaven to a full beard (in two participants), but most targets had a comparable style of short stubble. Participants were seated on a barstool 0.5 m from a plain white background. They were asked to sit straight with hands hanging freely alongside their bodies, look directly into the camera, and adopt a neutral expression. The camera (a sensor plane, marked φ) was positioned 125 cm from the participant and its height adjusted individually for each target to centre his head in the middle of the frame [distance between the camera and the participant was checked with a digital laser rangefinder (Bosch PLR 15)]. This setting of camera distance, focal length, and sensor size yielded a 35 × 53 cm field of view (23.85° angle of view).

### Post-processing of photographs

Image processing was carried out in Adobe Lightroom Classic CC (version 2017) and Adobe Photoshop CC 2015. We converted the images into DNG raw files and created DNG colour calibration profiles (using the X-Rite Color Checker Passport Lightroom plugin). Then we applied the profiles to all photographs. The calibrated images were exported into 16-bit Adobe RGB TIFF files in their actual size (35 × 53 cm) with a 168 PPI resolution. We manually checked the exposure (using the eye-drop tool on the background above the participants’ heads) and corrected the exposition on 85% value of every channel in the RGB colour space if necessary. Horizontal and vertical position of each participant in the image was adjusted using the Lightroom Transform tool (target’s head was positioned into the centre of the frame with pupils on a horizontal line). Then we batch-cropped to fit the heads on 27″ monitors in 1:1 size.

In the next step, we removed any possible disturbing creases or shadows in the background. Finally, we converted the photographs into an sRGB colour space and exported them into an 8-bit JPEG format (2101 × 3031 resolution, 168 PPI, sRGB) for the rating.

### Measurements of facial skin colour

#### In vivo measurements with a spectrophotometer

Facial skin colour was measured in vivo with a spectrophotometer Ocean Optics Flame-S with optical resolution of 2 nm using a standard D65 illuminant. Integrating Sphere ISP-R was used to spatially integrate the radiant flux in scatter transmission and diffuse reflectance sample measurements. The spectrophotometer was calibrated using the WS-1 Diffuse Reflectance Standard. All measurements were taken on three patches of targets’ faces (forehead, left and right cheek) and expressed also in CIE L*a*b* colour space^67^.

#### Facial photographs

We have also measured facial skin colour from the calibrated pre-vaccination facial photographs using ImageJ software (v 1.51) and Color Transformer 2 MatLab package. We measured the skin colour in CIE L*a*b* colour space and recorded the values for facial redness (a*), yellowness (b*), and lightness (L*) in three places of the face (forehead, right and left cheek)^68^. We measured the largest available area per stimulus while avoiding freckles, blemishes, and hair whenever possible. Facial skin colour values obtained from the spectrophotometer and from the facial photographs in our sample correlated positively (right cheek L* ρ = 0.314, left cheek L* ρ = 0.271, forehead L* ρ = 0.458; right cheek a* ρ = 0.271, left cheek a* ρ = 0.187, forehead a* ρ = 0.442; right cheek b* ρ = 0.685, left cheek b* ρ = 0.496, forehead b* ρ = 0.250). To facilitate a comparison with previous studies, we decided to use in further analyses in the main text facial skin colour measurements from the photographs. The results of analyses using spectrophotometer can be found in the Supplementary Materials—Tables S1, S2, S3, S4, S5, S6, S7, S8 and S9.

### Skin patches

We cropped skin patches from the obtained facial photographs in Adobe Photoshop CC 2015. The area of skin patches (89 × 89px) and location from which they were acquired (left cheek and forehead) were standardised while making sure that the resulting patch did not include any facial features (eyes, nose), hair, or birthmarks. The resulting skin patches (left cheek N = 21; forehead N = 18, in three instances the hair was covering the foreheads and we were unable to find any suitable patch) were enlarged by 300% for subsequent presentation (as per Jones et al.^69^).

**Figure 1:**
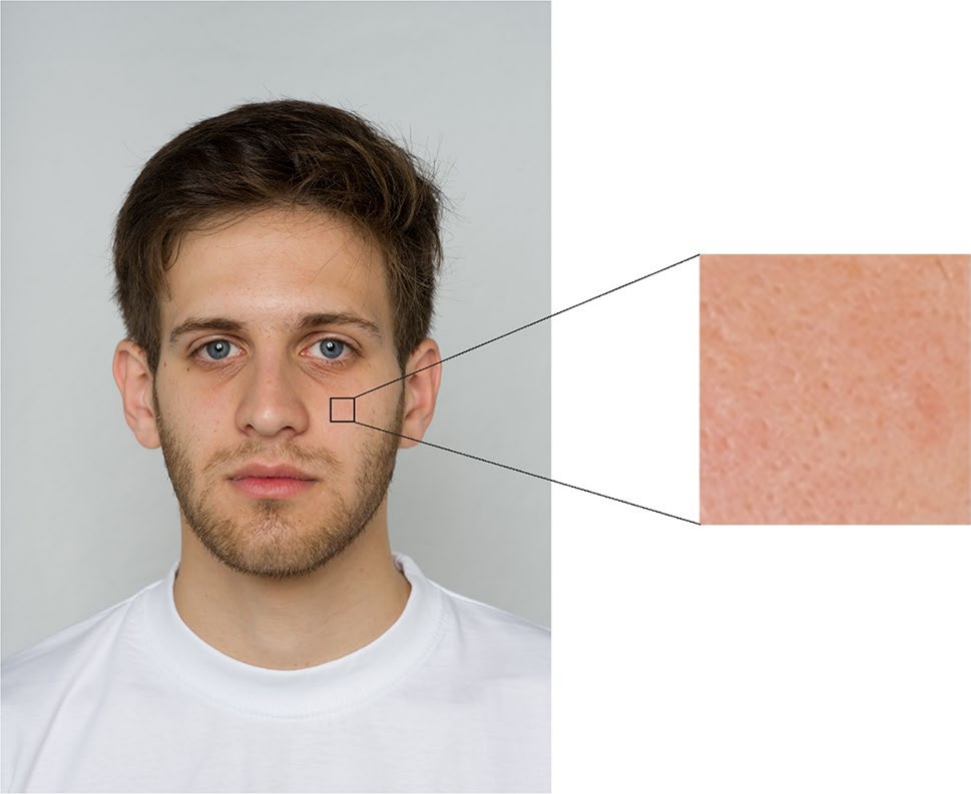
An example of acquired facial photograph with an outlined skin patch on the left and the resulting skin patch on the right (informed consent was obtained to publish the image in an online open-access publication).

A sample of portrait with outlined skin patch can be found in Fig. 1.

### Raters

Raters were recruited via social media sites (Facebook), oral invitations, and posters in university halls of the Faculty of Science, Faculty of Humanities, and the Faculty of Physical Education and Sport (all Charles University, Prague, Czechia). In Study 1, facial photographs were rated by 88 females aged 18–40 (mean = 22.87 ys; SD = 2.85) during Q1 2018. The raters received a reimbursement of 200 CZK (app. €8) as a compensation for their participation in the whole project (which also included ratings of voice recordings and body odour).

In Study 2, the obtained photographs and skin patches were rated by 62 females aged 18–40 (mean = 22.6 ys; SD = 3.42) during Q1 2019. The raters received a reimbursement of 50 CZK (app. €2) as a compensation for their time.

In Study 3, the photographs were rated by 66 females aged 18–40 (mean = 23 ys; SD = 4.71) during Q4 2019. They received a reimbursement of 150 CZK (app. €6) as a compensation for their participation in a larger rating session unrelated to the current investigation.

None of the raters in the three studies used hormonal contraception.

### The rating procedure

Study 1 was part of a larger project that also included the rating of voice recordings and body odour samples^53^. Exposure to a higher number of odour samples increases the risk of olfactory adaptation and can therefore affect rating, which is why the rating sessions were conducted on two separate days to accommodate a larger number of raters. From the total of 88 raters, 43 took part on the first day and 45 on the second day, which corresponds to the number of raters per photograph (depending on which day the photograph was presented). Randomly selected half of pre-vaccination samples was presented on the first rating day and half of post-vaccination photographs was presented on day two and vice versa. All raters were assessing photographs only once within a single day.

Study 2 was carried out using same rating procedure to eliminate any possible effects of a different data collection design. Of the 62 raters, 32 rated the first half of the randomly selected stimuli and 30 the second half of the stimuli. For Study 3, the procedure was identical and of the 66 raters, 31 took part on the first data collection day, 35 on the second day.

All rating (Study 1–3) took place in the Human Ethology perception lab under standardised conditions across all raters and rating days (closed window blinds, with artificial lighting to eliminate any changes in ambient lighting). The rating was conducted using Qualtrics survey suite (Qualtrics, Provo, UT) on two desktop computers of identical configuration with colour and brightness calibrated (by X-Rite i1Display Pro probe) LCD monitors (27″ Dell U2718Q UltraSharp IPS; 3840 × 2160 @ 168 DPI, 99% sRGB colour space coverage) turned to a vertical position to accommodate life-sized facial images.

The raters were seated 115 cm from the screen with eyes at a height of 125 cm (measured from the floor to the outer corner of the eye). This is a height and distance comparable to that from which the portrait photographs were taken, whereby raters were positioned into the same centre of projection and eye level. This setup approximates the common interpersonal distance^65,70^. Photographs were presented in randomised order.

In Study 1, all facial photographs (N = 21) were rated for attractiveness on a 7-point verbally anchored scale. In Study 2, participants rated portrait photographs (N = 21) on a 7-point verbally anchored scale for attractiveness again to check the robustness of acquired ratings. They also rated skin patches from left cheek (N = 21) and forehead (N = 18) on a 7-point verbally anchored scale regarding their healthiness. Due to a low number of forehead patches (hair in the images), we use only cheek patches in analysis below. In Study 3, portrait photographs (N = 21) were rated on a 7-point verbally anchored scale regarding healthiness.

After rendering their rating assessments (Study 1–3), raters completed a questionnaire about their basic demographics (age, education, occupation, etc.).

### Data analyses

To determine the consistency of raters’ assessments, we performed an intra-class correlation (ICC) analysis for each group rating the same set of samples using IBM SPPSS Statistics (v 23). All remaining statistical analyses were performed in jamovi (v 1.6.15).

To explore relationships between variables, ratings of facial attractiveness from Study 1 and 2, and facial healthiness from Study 3, we used Spearman’s rank correlation coefficient because the data deviated from normal distribution. We set ρ ≥ 0.8 as a value at which we would consider the two variables highly correlated. In such case, only one of the variables would be used for subsequent analyses^71^. Further, we used the Spearman’s rank correlation to test the association between levels of antibodies and targets’ age.

We used a one-way analysis of variance (ANOVA) with Tukey post-hoc test to test for differences and Spearman’s rank correlation coefficient for strength of associations between separate colour measurements from the right and left cheek and the forehead.

To examine the relationship between perceived facial attractiveness (Study 1), perceived skin patch healthiness (Study 2), perceived facial healthiness (Study 3), and differences in antibody levels (pre-vaccination subtracted from 30 days post-vaccination), we specified three separate linear mixed-effects models (LMMs) using the GAMLj module in jamovi. The rated characteristics (facial attractiveness, healthiness, and skin patch healthiness) were entered as dependent variables, while differences in antibodies against hepatitis A (Anti-Hav) and meningococcus (Anti-Mnk) were entered as predictors. To control for variability in targets and raters, we entered the targets’ and raters’ IDs as random effects (example of the model entry: Facial Attractiveness ~ 1 + State/antibody levels/ + (1|ID_rater) + (1|ID_donor)). We employed analogous models to assess the relationships between the rated characteristics (facial attractiveness and healthiness), steroid hormones levels, and the percentage of adipose tissue, and to assess the relationship between the rated characteristics (facial attractiveness, healthiness, and skin patch healthiness) and forehead and cheek lightness, redness, and yellowness. To explore a possible relationship between targets’ age and perceived facial attractiveness and healthiness, we ran analogous separate linear mixed-effects models, with the rated characteristic entered as a dependent variable and age as the predictor.

To test the association between differences in antibody levels (pre-vaccination and 30 days post-vaccination), basal levels of steroid hormones (testosterone and cortisol), and the percentage of adipose tissue, we employed general linear models (GLM) using the GAMLj jamovi module. In both models, we entered specific antibodies (Anti-HAV or Anti-Mnk) as dependent variables and steroid hormones and percentage of adipose tissue as predictors (e.g., Anti-HAV ~ 1 + basal cortisol + basal testosterone + adipose tissue (%)). Analogous tests were carried out to investigate the relationship between antibody levels (pre- and 30 days post-vaccination) and forehead and cheek lightness, redness, and yellowness. For information about model residuals, see Supplementary Materials S1.

We performed a simulation-based power analysis for each fixed-effect predictor in our LMMs^72^ to estimate observed power using the SimR package^73^ in R (for a discussion of limits of observed power, see Lakens^74^). Further, based on simulated data (gradually increasing the sample size to 100), we plotted Power curves showing the sensitivity to detect observed effects with α = 0.05. The results of observed power, Power curve plots, and the R script are available in the Supplementary Materials S2, S3.

## Results

Descriptive statistics for targets’ basic demographic data, rated characteristics, differences between pre- and 30 days post-vaccination antibody levels, steroid hormone levels, the percentage of adipose tissue, and colour measurements are presented in Table 1. For detailed information, see Table S10 in Supplementary Materials.

**Table 1:** Descriptive statistics for target’s age, height and weight, ratings of facial photographs and skin patches before vaccination, specific antibodies (difference between states 30 days after and before vaccination), testosterone and cortisol basal levels, the amount of adipose tissue, and facial skin colour (L*a*b* for cheeks and forehead) (N = 21).

Parameter name	Mean	SD	Range
Age (ys)	26.19	4.62	20–35
Height (cm)	181	6.74	169–198
Weight (kg)	78.9	14.8	58.5–130
Facial attractiveness S1	3.08	0.978	1.37–4.63*
Facial attractiveness S2	3.18	0.772	1.75–4.38*
Cheek patch healthiness S2	3.75	0.803	2.69–5.4*
Facial healthiness S3	4.38	0.932	2.23–5.91*
Anti-HAV antibodies (arb. U.)	− 1.07	1.01	− 2.11–1.55
Anti-Mnk antibodies (IU/I)	14.6	17.4	0.14–56.4
Basal testosterone (ug/l)	4.33	1.23	2.25–7.1
Basal cortisol (nmol/l)	471	91.4	282–662
Adipose tissue (%)	17.5	6.86	5.00–32.8
Left cheek lightness	67.9	2.83	63.7–74.2
Right cheek lightness	69	2.45	65.8–76.3
Forehead lightness	74.1	2.9	66.4–80.1
Left cheek redness	12.7	1.75	9.41–15.8
Right cheek redness	12.5	1.79	9.78–15.8
Forehead redness	10.2	1.77	6.54–14.7
Left cheek yellowness	18.2	2.54	14.2–23.7
Right cheek yellowness	17.3	2.16	14.5–22.1
Forehead yellowness	16.2	2.47	12.5–20.8

Mean (SD) rating for facial photographs and skin patches was calculated as the mean from aggregated ratings for each target. Values denoted by * show the mean minimum and mean maximum ratings of photographs.

We found high level of agreement between raters in all rated characteristics (ICC above 0.864). For further details, see Table S11 in Supplementary Materials.

### Relationships between variables

Ratings of facial attractiveness collected in Study 1 and 2 were strongly positively and statistically significantly correlated (ρ = 0.937, *p* < 0.001). In all subsequent analyses, we therefore use attractiveness ratings from Study 1.

Ratings of perceived facial attractiveness (Study 1) and perceived healthiness (Study 3) were also positively and statistically significantly correlated (ρ = 0.706, *p* < 0.001). The value of ρ did not, however, reach the pre-set level of 0.8, and we therefore analyse perceived facial attractiveness and healthiness separately.

Linear mixed-effects model testing the relationship between targets’ age and perceived facial attractiveness (R^2^_C_ = 0.523, R^2^_M_ = 0.030) did not show a statistically significant association (β = − 0.063 [− 0.154, 0.028], *p* = 0.193). The relationship between perceived facial healthiness and targets’ age (R^2^_C_ = 0.474, R^2^_M_ = 0.035) was likewise not statistically significant (β = − 0.069 [− 0.155, 0.016], *p* = 0.130). Further, we found no statistically significant relationship between antibody levels and targets’ age (ρ_Anti-HAV_ = − 0.130, *p* = 0.573; ρ_Anti-Mnk_ = − 0.078, *p* = 0.738). In subsequent analyses, we therefore did not control for age.

Left and right cheek measures of skin lightness (ρ = 0.801, *p* < 0.001), redness (ρ = 0.861, < 0.001), and yellowness (ρ = 0.925, *p* < 0.001) were strongly positively and statistically significantly associated. We thus continue to use only L* a* b* measures from the left cheek in further analyses because we presented the left cheek patches to participants in Study 2 for patch healthiness ratings.

Skin lightness (ρ = 0.444, *p* = 0.044), redness (ρ = 0.544, *p* = 0.011), and yellowness (ρ = 0.689, *p* < 0.001) from the left cheek and forehead were also positively and statistically significantly correlated. The ρs did not, however, reach the predefined level (0.8) and we therefore use the left cheek and forehead measures in further analyses separately. For further details, see Table S12 in Supplementary Materials.

**Figure 2:**
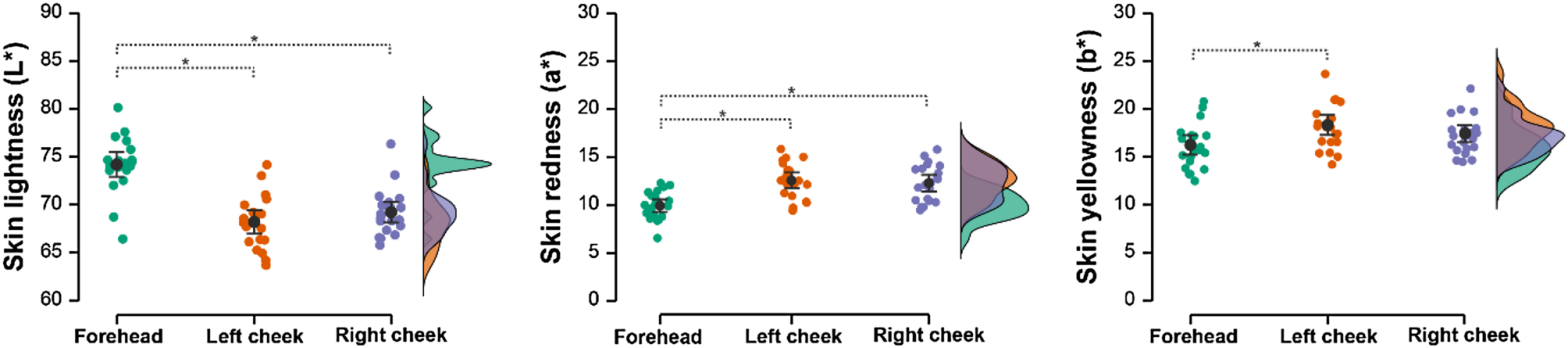
Differences in skin colour (CIE L*a*b*) measured from right and left cheek and the forehead. Black dots represent mean values, error bars show their 95% confidence intervals. Coloured points represent individual data points, while density plots show their distribution. Statistically significant differences are marked by asterisk.

In our targets, skin on the forehead was statistically significantly lighter (L*) and statistically significantly less red (a*) and less yellow (b*) than skin on either cheek (for skin yellowness (b*), there was a statistically significant result for forehead and left cheek only). The two cheeks did not differ significantly in either L*, a*, or b* measures (see Fig. 2 and Tables S13–S15 in Supplemental Materials).

### Study 1: association between perceived facial attractiveness, antibody levels, colouration, and immunomodulatory factors

Linear mixed-effects models show that perceived facial attractiveness (R^2^_C_ = 0.532, R^2^_M_ = 0.024) was not predicted by levels of specific antibodies. For details, see Table 2.

**Table 2:** Relationship between reactivity of the immune system and perceived facial characteristics.

Characteristic	Predictors	F	β	95% CI (LL, UL)	df	t	SE	*p*
Facial attractiveness	Anti-HAV	0.955	0.217	− 0.218, 0.651	18	0.977	0.222	0.341
	Anti-Mnk	0.568	0.010	− 0.016, 0.035	18.2	0.753	0.013	0.461
Facial healthiness	Anti-HAV	0.140	− 0.080	− 0.501, 0.340	18.2	− 0.375	0.215	0.712
	Anti-Mnk	0.755	0.011	− 0.014, 0.035	18.5	0.869	0.013	0.396
Cheek patch healthiness	Anti-HAV	0.244	− 0.089	− 0.443, 0.265	18.4	− 0.494	0.181	0.627
	Anti-Mnk	1.593	0.013	− 0.007, 0.034	18.8	1.262	0.011	0.222

Attractiveness ratings: for target ID, VRC = 0.960, SD = 0.980, ICC = 0.429; for rater ID, VRC = 0.429, SD = 0.655, ICC = 0.251. Relationship between perceived healthiness and reactivity of the immune system. Facial healthiness ratings: for target ID, VRC = 0.876, SD = 0.936, ICC = 0.374; for rater ID, VRC = 0.458, SD = 0.677, ICC = 0.238. Relationship between perceived cheek patch healthiness and reactivity of the immune system. Cheek patch healthiness ratings: for target ID, VRC = 0.608, SD = 0.780, ICC = 0.324; for rater ID, VRC = 0.487, SD = 0.698, ICC = 0.278.

*Anti-HAV* antibodies against hepatitis A, *Anti-Mnk* antibodies against meningococcus, *VRC* variance of random components.

 Neither redness, yellowness, nor lightness of the forehead or left cheek predicted elevations in any of the specific antibodies (Anti-HAV: R^2^ = 0.447, R^2^_adj_ = − 0.210; Anti-Mnk: R^2^ = 0.350, R^2^_adj_ = 0.071). For detailed results, see Table 3.

**Table 3:** Relationship between reactivity of the immune system and forehead and cheek lightness, redness, and yellowness.

Characteristic	Predictors	F	β	95% CI (LL, UL)	df	t	SE	*p*
Anti-HAV	Left cheek lightness	1.157	0.372	− 0.132, 0.396	14	1.075	0.123	0.300
	Forehead lightness	0.022	0.063	− 0.296, 0.340	14	0.147	0.148	0.885
	Left cheek redness	3.197	0.542	− 0.062, 0.686	14	1.788	0.175	0.095
	Forehead redness	1.060	− 0.314	− 0.552, 0.194	14	− 1.029	0.174	0.321
	Left cheek yellowness	3.439	0.936	− 0.058, 0.800	14	1.854	0.200	0.085
	Forehead yellowness	0.040	− 0.116	− 0.556, 0.462	14	− 0.200	0.237	0.845
Anti-Mnk	Left cheek lightness	0.334	− 0.216	− 6.26, 3.60	14	− 0.578	2.30	0.573
	Forehead lightness	0.555	0.345	− 3.88, 8.01	14	0.745	2.77	0.468
	Left cheek redness	0.376	0.202	− 5, 9	14	0.613	3.26	0.550
	Forehead redness	2.283	− 0.500	− 11.89, 2.06	14	− 1.511	3.25	0.153
	Left cheek yellowness	0.284	− 0.292	− 10.02, 6.03	14	− 0.533	3.74	0.602
	Forehead yellowness	0.115	0.214	− 8.01, 11.02	14	0.339	4.44	0.740

*Anti-HAV* antibodies against hepatitis A, *Anti-Mnk* antibodies against meningococcus. β represents a standardized β estimate.

Running a linear mixed-effects model, we found that neither the levels of cortisol or testosterone, nor the percentage of adipose tissue predicted perceived facial attractiveness (R^2^_C_ = 0.534, R^2^_M_ = 0.099). For details, see Table 4.

**Table 4:** Relationship between perceived facial attractiveness and healthiness, cortisol, testosterone levels, and adipose tissue.

Characteristic	Predictors	F	β	95% CI (LL, UL)	df	t	SE	*p*
Facial attractiveness	Cortisol	1.445	− 0.003	− 0.008, 0.002	17.1	− 1.202	0.002	0.246
	Testosterone	0.136	0.085	− 0.366, 0.536	17	0.368	0.230	0.717
	Adipose tissue	3.023	− 0.075	− 0.160, 0.010	17	− 1.739	0.043	0.100
Facial healthiness	Cortisol	5.265	− 0.005	− 0.010, − 0.001	17.3	− 2.295	0.002	0.035
	Testosterone	1.019	0.223	− 0.210, 0.657	17	1.010	0.221	0.327
	Adipose tissue	0.348	− 0.025	− 0.106, 0.057	17.1	− 0.590	0.042	0.563

Attractiveness ratings: for target ID, VRC = 0.763, SD = 0.873, ICC = 0.374; for rater ID, VRC = 0.429, SD = 0.655, ICC = 0.251. Relationship between facial healthiness and cortisol, testosterone levels, and percentage of adipose tissue.

Facial healthiness ratings: for target ID, VRC = 0.690, SD = 0.831, ICC = 0.320; for rater ID, VRC = 0.458, SD = 0.676, ICC = 0.238. *VRC* variance of random components.

In a GLM analysis, neither the levels of testosterone or cortisol, nor the percentage of adipose tissue predicted elevations in any of the specific antibodies Anti-HAV (R^2^ = 0.084, R^2^_adj_ = − 0.078) and Anti-Mnk (R^2^ = 0.186, R^2^_adj_ = 0.042). For detailed results, see Table 5.

**Table 5:** Relationship between reactivity of the immune system, steroid hormones levels, and adipose tissue.

Characteristic	Predictors	F	β	95% CI (LL, UL)	df	t	SE	*p*
Anti-HAV	Cortisol	1.101	0.270	− 0.003, 0.009	17	1.049	0.003	0.309
	Testosterone	0.170	− 0.136	− 0.682, 0.458	17	− 0.413	0.270	0.685
	Adipose tissue	0.100	− 0.109	− 0.123, 0.091	17	− 0.317	0.051	0.755
Anti-Mnk	Cortisol	0.433	− 0.160	− 0.128, 0.067	17	− 0.658	0.046	0.520
	Testosterone	3.158	0.551	− 1.461, 17.07	17	1.777	4.392	0.093
	Adipose tissue	1.928	0.452	− 0.594, 2.883	17	1.389	0.824	0.183

*Anti-HAV* antibodies against hepatitis A, *Anti-Mnk* antibodies against meningococcus. β represents a standardized β estimate.

In a linear mixed-effects model testing the influence of skin colour on perceived facial attractiveness (R^2^_C_ = 0.540, R^2^_M_ = 0.190), forehead redness was the only statistically significant predictor with a negative slope (β = − 0.490 [− 0.780, − 0.201]); see Table 6.

**Table 6:** Relationship between perceived characteristics and facial colouration.

Characteristic	Predictors	F	β	95% CI (LL, UL)	df	t	SE	*p*
Facial attractiveness	Left cheek lightness	0.013	0.012	− 0.197, 0.222	15.5	0.114	0.107	0.911
	Forehead lightness	0.496	− 0.090	− 0.340, 0.160	14.8	− 0.704	0.127	0.492
	Left cheek redness	0.352	0.090	− 0.206, 0.386	15.2	0.593	0.151	0.562
	Forehead redness	11.038	− 0.490	− 0.780, − 0.201	14.1	− 3.322	0.148	0.005
	Left cheek yellowness	1.865	0.232	− 0.101, 0.567	14.3	1.366	0.171	0.193
	Forehead yellowness	0.159	− 0.081	− 0.476, 0.315	14.2	− 0.399	0.202	0.696
Facial healthiness	Left cheek lightness	3.895	0.188	0.001, 0.375	14.2	1.974	0.095	0.068
	Forehead lightness	2.671	− 0.188	− 0.414, 0.038	14.2	− 1.634	0.115	0.124
	Left cheek redness	0.065	0.034	− 0.229, 0.298	13.9	0.254	0.135	0.803
	Forehead redness	7.023	− 0.357	− 0.620, − 0.093	14.1	− 2.650	0.135	0.019
	Left cheek yellowness	0.867	0.144	− 0.159, 0.447	14	0.931	0.155	0.368
	Forehead yellowness	0.182	− 0.078	− 0.436, 0.280	13.9	− 0.427	0.183	0.676
Cheek patch healthiness	Left cheek lightness	3.035	0.112	− 0.014, 0.238	17.1	1.742	0.064	0.099
	Left cheek redness	7.313	− 0.240	− 0.414, − 0.066	16.5	− 2.704	0.089	0.015
	Left cheek yellowness	0.013	0.007	− 0.110, 0.123	16.6	0.113	0.059	0.911

Facial attractiveness ratings: for target ID, VRC = 0.545, SD = 0.739, ICC = 0.299; for rater ID, VRC = 0.429, SD = 0.655, ICC = 0.251. Facial healthiness ratings: for target ID, VRC = 0.433, SD = 0.658, ICC = 0.228; for rater ID, VRC = 0.458, SD = 0.677, ICC = 0.238. Cheek patch healthiness ratings: for target ID, VRC = 0.244, SD = 0.493, ICC = 0.161; for rater ID, VRC = 0.490, SD = 0.7, ICC = 0.279. *VRC* variance of random components.

### Study 2: association between perceived cheek patch healthiness and colouration

A linear mixed-effects model shows that perceived cheek patch healthiness (R^2^_C_ = 0.478, R^2^_M_ = 0.025) was not predicted by levels of specific antibodies (Table 2). In a linear mixed-effects model testing the influence of skin colour on perceived cheek patch healthiness (R^2^_C_ = 0.471, R^2^_M_ = 0.164), cheek redness negatively predicted perceived cheek patch healthiness. For detailed information, see Table 6.

### Study 3: association between perceived facial healthiness, antibody levels, colouration, and immunomodulatory factors

A linear mixed-effects model shows that perceived facial healthiness (R^2^_C_ = 0.484, R^2^_M_ = 0.015) was not predicted by levels of specific antibodies (for details, see Table 2). In a separate linear mixed-effects model testing the influence of skin colour on perceived facial healthiness (R^2^_C_ = 0.491, R^2^_M_ = 0.182), forehead redness negatively predicted (β = − 0.357 [− 0.620, − 0.093]) perceived facial healthiness (see Table 6), though we stress out the effect’s 95% CIs span from substantially negative (LL = − 0.620) to negligible ones (UL = − 0.093).

A linear mixed-effects model (R^2^_C_ = 0.487, R^2^_M_ = 0.086) testing the association between cortisol, testosterone, adipose tissue percentage, and facial healthiness shows that only cortisol levels marginally negatively predicted (β = − 0.005 [− 0.010, − 0.001]) perceived facial healthiness. For details, see Table 4.

## Discussion

The main aim of all three studies was to test for possible associations between immune system reactivity (an organism’s ability to effectively respond to an antigen) and perceived attractiveness and healthiness. We found no statistically significant associations between experimentally elicited levels of antibodies against hepatitis A (Anti-HAV) or meningococcus (Anti-Mnk) and perceived facial attractiveness, healthiness, or healthiness of skin patches. Moreover, we observed no statistically significant associations between the levels of antibodies and testosterone, cortisol, or adipose tissue, which are all variables often associated with immune function. Adipose tissue and testosterone and cortisol levels also showed no connection with perceived facial attractiveness. Notably, we found a small negative effect of cortisol levels on perceived facial healthiness. Further, we found that higher forehead redness was perceived as less attractive and healthy when individuals assessed portrait photographs, and for cheek patches, higher cheek redness was perceived as less healthy. No systematic relationship was found between measures of facial skin colouration and Anti-HAV and Anti-Mnk antibodies.

We examined possible relationships between immunoreactivity and facial attractiveness and healthiness because it has often been claimed that attractive traits are related to underlying qualities of individuals. Previous studies indeed reported a positive link between male facial attractiveness and either cytokine levels after stimulation with LPS (r = 0.291, N = 41)^7^ or elevated immune system response to vaccination against hepatitis B (β = 0.5, N = 74)^9^. Although the 95% confidence intervals of our results regarding the association between Anti-HAV, Anti-Mnk, and perceived facial attractiveness (β_anti-HAV_ = 0.217, [− 0.218, 0.651]; β_anti-MNK_ = 0.01, [0.016, 0.035]; N = 21) do partially overlap with results of some studies that found significant relation between perceived facial attractiveness and hepatitis B antibodies levels after vaccination^9^, our results are more in line with the studies by Skrinda et al.^11^ and Rantala et al.^10^ who found no support for significant associations between hepatitis B antibody levels after vaccination and perceived attractiveness in men (β = − 0.21, N = 60) and women (r = − 0.006, N = 52), respectively. Overall, as noted at the outset, empirical evidence regarding an association between immunocompetence and facial attractiveness remains equivocal^75^.

The strength of our study lies in using vaccines against both viral (hepatitis A and B) and bacterial (meningococcus) diseases. This way, we aimed to stimulate a wider array of immune system components because, for example, the advantage of heterozygotic individuals is the greatest when they fight against multiple pathogens at once^76^. Moreover, we inspected both immunoreactivity and facial colouration. Unlike some previous studies^9,50^, we excluded hepatitis B antibodies from our analyses because several participants showed high levels of the relevant antibodies already in the baseline measurement, while others did not react to the vaccine.

In general, the use of vaccination to stimulate immunoreactivity has some limitations. For the purpose of this study, we treated a higher level of antibodies as a proxy to higher disease resistance. This is, however, something of a simplification because higher immunoreactivity is not always adaptive^77^. Excessively strong (hypersensitivity) or inappropriate (e.g., autoimmune) immunity response is not beneficial and can ultimately negatively affect individual fitness. Moreover, by focusing solely on antibody levels, one can only arrive at generalised and limited information about the function of the immune system. Investigation of differences of the immune response in its humoral and cellular components and of the trade-offs between them might provide a more nuanced insight.

Recent studies employed several methods of measuring the functioning of the immune system and arrived at rather diverse results, making our null results no exception. Foo et al.^78^ focused on innate immunity and measured salivary immune function (antibacterial capacity against *Escherichia coli* and lysosome activity against *Micrococcus lysodekticus*) alongside oxidative stress and semen quality. Using principal component analyses, they obtained two factors: PC1—bacterial-killing capacity and overall bacterial immunity and PC2—bacterial suppression capacity and lysozyme activity. Contrary to expectations, no connection was found between the selected physiological measures of immune function, attractiveness (r_PC1_ = − 0.16, r_PC2_ = 0.04, N = 98) and number of sexual partners (r_PC1_ = − 0.07, r_PC2_ = − 0.10, N = 97)^78^. Phalane et al.^7^, on the other hand, found a positive relationship between cytokine levels (after stimulation of the immune system with LPS) (r = 0.291, N = 41) and male facial attractiveness but not the CRP (r = − 0.085, N = 41)^7^. Cai et al.^79^ employed as a marker of immune function salivary immunoglobulin A (IgA), which acts as a defence against microbial invasion. They found no connection between IgA and female facial attractiveness (ρ = − 0.051, N = 221)^79^.

It has been proposed that facial skin provides information about the functioning of the immune system and about health^16,69,80^. It has been demonstrated that people can assess health and attractiveness even from limited information such as skin patch and their ratings correspond to their ratings of the whole face^15,69^. In our study, we therefore used cheek skin patches to limit possible effects of confounding factors (e.g., face shape). We also investigated relationships between perceived healthiness of the skin patch and direct measures of immune system function. And yet, we found no associations between perceived skin healthiness and levels of specific antibodies.

Previous studies reported that testosterone and cortisol have an effect on both the functioning of the immune system and perceived facial attractiveness and healthiness, and might thus work as mediators between the functioning of the immune system and perceived facial characteristics. According to the hypothesis of immunocompetence handicap, androgens exert immunosuppressive effects and only high-quality individuals (including their immunity) can produce and maintain a high level of testosterone and afford the physiological costs of lowered immunosuppression^40^. Although the results of some studies do support the hypothesis of immunosuppressive effects of sex hormones^38,81^, the overall pattern in literature is rather mixed (see, e.g., a metanalysis^41^). It has been suggested that glucocorticoids contribute to this complex picture because they modulate immune system response as well as the expression of secondary sexual characteristics, and they may interact with testosterone^42,43,45^. In our study, we found no significant effects of either testosterone or cortisol on antibody levels. This finding is consistent with the results of Nowak et al.^82^, who found no influence of testosterone on the effectiveness of immune system using the influenza vaccine. In contrast, though, in vitro studies did find an immunosuppressive effect of testosterone on a spontaneous production of IgG in mononuclear cells of human peripheral blood^83,84^. Rantala et al.^9^, however, showed that the immune system’s reactivity was higher in males with higher testosterone levels who simultaneously exhibited lower cortisol levels and, moreover, these males were perceived as more attractive by women. In our study, we found none of the expected associations between testosterone and the rated characteristics. We found only a weak negative association between cortisol and perceived healthiness, but not attractiveness. Interestingly, another study showed a negative association between attractiveness (but not healthiness) and cortisol levels^45^.

Additionally, we found that the two scales of facial attractiveness and healthiness are positively correlated but the magnitude of this association is not strong enough to treat the two as interchangeable. It is thus possible that facial attractiveness and healthiness stand for two separate perceptual qualities. This idea finds further support both in the negative association between cortisol levels and perceived facial healthiness reported in our study and in the negative association between cortisol levels and perceived attractiveness in a study by Moore et al.^45^. This suggests that one should exercise caution when selecting specific characteristics to be rated for individual studies.

It has been reported that higher adiposity contributes to reduced immunocompetence, and possibly impaired immune function accompanied by changes in leukocyte counts, lower antibody production, as well as worse wound healing and higher risk of infections^47,48^ Moreover, the faces of obese and overweight individuals are perceived as less attractive^5,51^. Adiposity thus seems to underlie the relationship between immune response and attractiveness^50^. In our study, we did not find any significant relationship between perceived facial attractiveness or healthiness and antibody levels or body fat percentage. One possible explanation might be that participants in our sample had a generally lower body fat percentage (mean = 17.5%): only two participants fell in the obese category with body fat percentage over 25% (threshold recommended by the American Council on Exercise). Our sample, where variability of body fat percentage was relatively low, may have been thus ill-suited to detecting the negative effect of increased adiposity. On the other hand, other studies detected a negative effect of higher weight (expressed by BMI) on immune function even within the range of average body weight variation^85^.

A number of previous studies reported associations between skin colour, facial appearance, and immune response. South African men with a higher cytokine response to stimulation (induced by LPS) had yellower, more ‘carotenoid’ skin colour^7^. Furthermore, yellower skin was preferred alongside lighter skin, but it is well possible that this preference for lighter skin is due to the yellow carotenoid colouration being more visible in lighter skin hues^7^. In our study, however, we found no statistically significant associations between skin yellowness and perceived characteristics. A number of other studies (e.g., Stephen et al.^17,24^) employed manipulation of skin colour in photographs, while we used natural portrait images. This may have resulted in a lower variability in our sample, thus potentially reducing the likelihood of observing the effect. Still, we found that both perceived facial attractiveness and healthiness were negatively predicted by higher forehead redness and cheek skin healthiness was negatively predicted by higher cheek redness. Although higher redness has been previously linked to higher perceived attractiveness and health^16,17^, the relationship need not be linear: it is possible that some level of redness may affect perceived attractiveness positively, but above a certain threshold it has a negative effect on perceived attractiveness^16^. We propose that higher (forehead) redness levels might be perceptually linked to dermatoses, such as rosacea^86^, acne, or other imperfections which are generally perceived as less attractive^80^.

Unlike various studies which measured skin colour from both cheeks and the forehead and averaged them into one value for facial skin lightness, redness, or yellowness^7,25,87^, we used facial skin colour measurements from the cheek and the forehead separately, as majority of colour measurements between those areas differed and were only moderately associated. Cheeks and forehead differ in the amount of subcutaneous fat and therefore also in blood perfusion, which might account for slight differences in colouration. Accordingly, it has been found that the variation of colour in different parts of the face matters, whereby for instance periorbital luminance, cheek redness, and overall yellowness of the face predict perceived health^88^. To some extent, though, the differences in the skin colour of various parts of the face in our sample might be also due to the methods we used for acquisition of facial photographs from which we measured the values of facial colours. Our aim was to simulate naturally occurring daylight conditions with a diffuse strobe light positioned above the participant’s head pointing downwards. In this setup, though, the light source was positioned relatively close. Due to inverse-square law of loss of light over distance, it may have reflected on the forehead, causing it to appear brighter than the cheeks, and it may have produced highlights responsible for the observed colour differences between forehead and cheeks. Further, we tried to limit any potential effects of bright spots and light reflections by patting participants’ foreheads with napkins and we waited for some time before taking the photographs to avoid any skin redness caused by this process. Still, some brighter areas may have appeared and caused specular highlights, thus affecting the measurements.

### Limitations

Aside from the limitations discussed above, the main limitation of the present study is the small sample size of targets (although comparable to some previous studies^89^), which resulted in wide confidence intervals of the effect sizes and a low power to observe the reported effects (in most cases below 50%). Based on our Power curve analysis, even a sample size of 100 targets would not yield a higher power (e.g., ≥ 80% for most effects).

We experienced significant obstacles in participant recruitment due to the relatively strict conditions for participation (we required not being vaccinated against either of the diseases of our interest in the past 10 years) as well as anti-vaccination biases which may have discouraged some individuals from participation^90^. Moreover, some participants showed high levels of antibodies against hepatitis B despite our entry requirement of not being recently vaccinated. Some participants may thus have been unaware of a relatively recent vaccination. Moreover, some participants did not respond to the hepatitis B vaccine, a phenomenon observed in app. 10% of population^91^. Therefore, we had to exclude antibodies against hepatitis B from our analyses. Note, that although hepatitis B vaccine is commonly used in other studies, vaccination against hepatitis B was in 2001 included in the compulsory vaccination protocol in Czechia. Consequently, when selecting a vaccine, the context of its use ought to be investigated more closely and a choice of vaccines against some less common diseases may be a better option.

Previous research has also suggested that immunoreactivity and cues to various aspects of immune system functioning may differ between the sexes^9,10^ and our results are based only on a male sample. Future investigations should thus include both men and women as targets and raters to better understand the complex relations between attractiveness and immunity and its role in intersexual selection.

## Conclusion

We investigated the relationship between functioning of the immune system and perceived facial attractiveness, healthiness, skin patch healthiness, and potential influence of skin colour. We employed measurements of antibodies after application of two different vaccines as markers of reactivity of the immune system and recorded the levels of steroid hormones (cortisol and testosterone) as well as the percentage of adipose tissue due to their immunomodulatory properties and connection to facial attractiveness. We found no significant relationships between reactivity of the immune system and perceived facial attractiveness, healthiness, and skin patch healthiness. We did, however, observe a small negative effect of cortisol on perceived facial healthiness. Moreover, steroid hormones and adipose tissue showed no relationship to either the immune response after vaccination or skin colouration. Finally, higher forehead redness from portrait photographs was perceived as both less attractive and healthy and higher cheek redness from skin patches was perceived as less healthy. Our results thus suggest that facial attractiveness and healthiness provide a limited amount of cues to immune system functioning and perceived characteristics seem to be related only to certain hormone levels and facial colour.

Despite some limitations, we believe that our study is a valuable contribution to research on the role of visual cues in assessments of functioning of the immune systems of individuals, and that it can serve as an entry for future meta-analysis aimed at disentangling the conflicting results of various existing studies. Future studies might also investigate the activation of different components of the immune system, such as humoral and cellular immunity, and focus on acquiring larger samples.

## Supplementary Information

Supplementary Information 1.

Supplementary Information 2.

Supplementary Information 3.

Supplementary Information 4.

Supplementary Information 5.

Supplementary Information 6.

Supplementary Information 7.

Supplementary Information 8.

Supplementary Information 9.

Supplementary Information 10.

## Data Availability

The data associated with this research are available at https://osf.io/4k3ud/.
