# Supplementary material for: Attractive and healthy-looking male faces do not show higher immunoreactivity
Source: Sci Rep. 2022 Nov 1;12:18432. doi: 10.1038/s41598-022-22866-x (PMC9626598; doi:10.1038/s41598-022-22866-x)

Relationship between perceived facial attractiveness and Anti-HAV and Anti-Mnk levels

Assumption Checks

Test for Normality of residuals

Test	Statistics	p
Kolmogorov-Smirnov	0.0383	0.134
Shapiro-Wilk	0.9929	< .001

Q-Q Plot

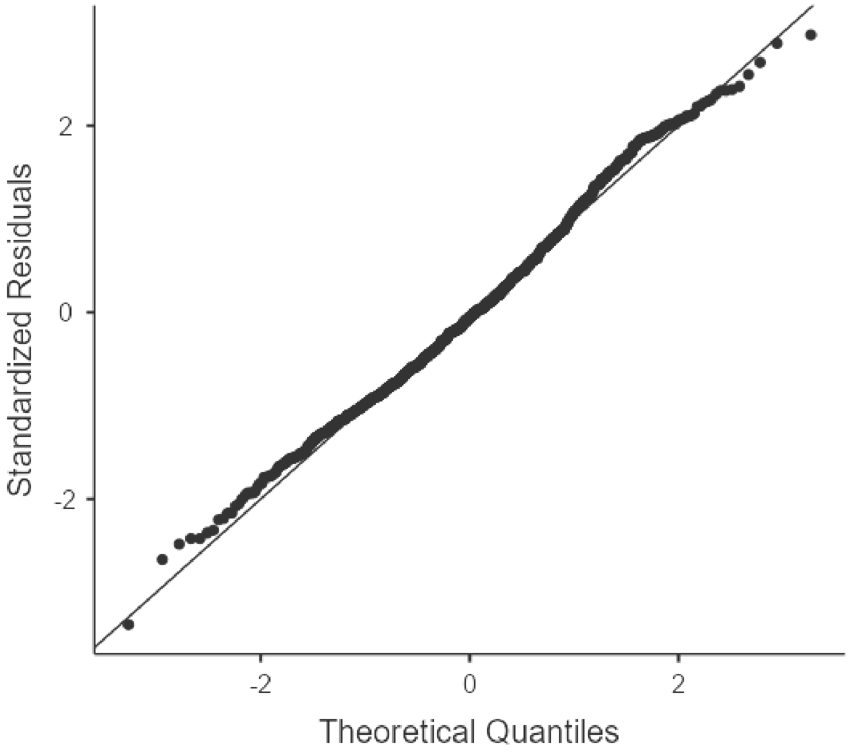

Residual histogram

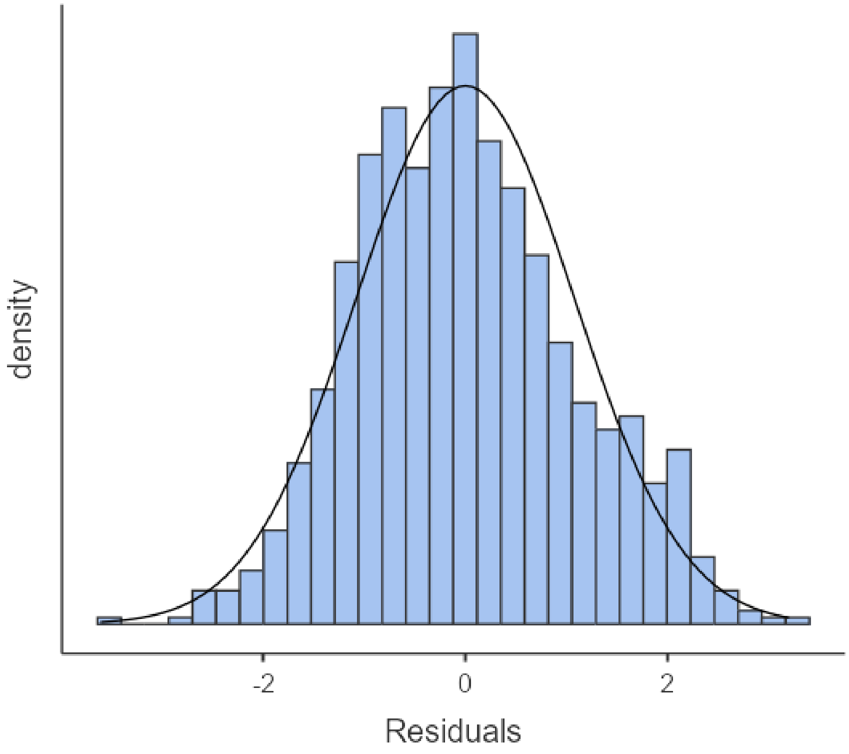

Relationship between perceived facial healthiness and Anti-HAV and Anti-Mnk levels

Assumption Checks

Test for Normality of residuals

Test	Statistics	p
Kolmogorov-Smirnov	0.0333	0.421
Shapiro-Wilk	0.9967	0.166

Q-Q Plot

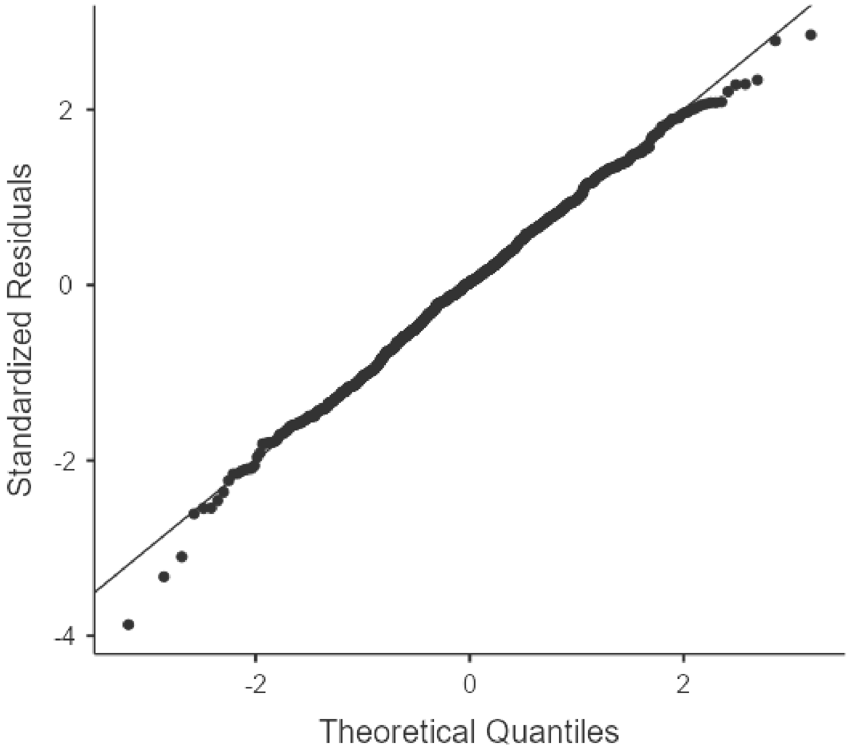

Residual histogram

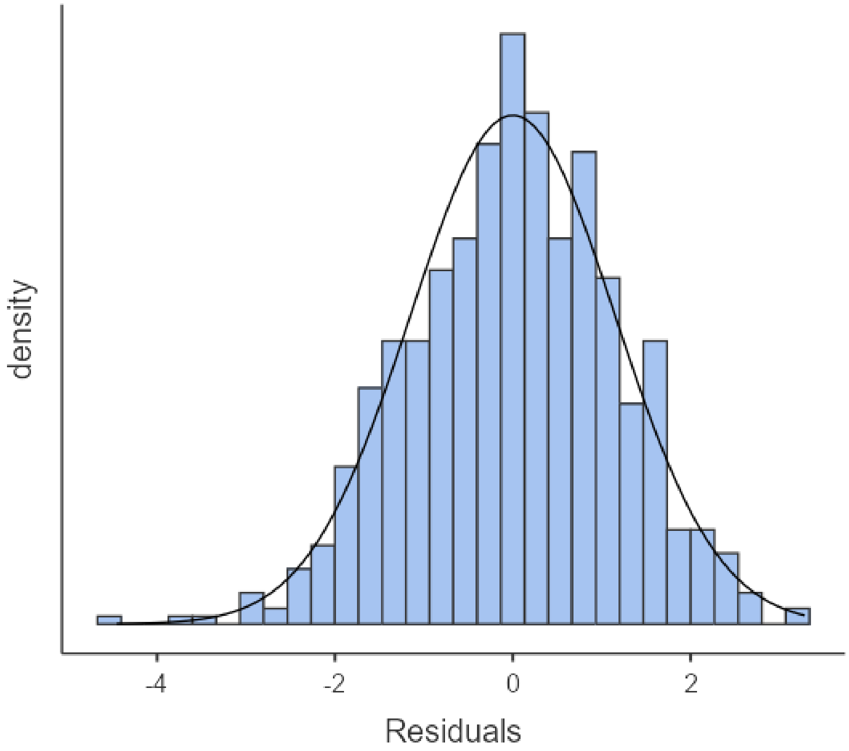

Relationship between perceived cheek patch healthiness and Anti-HAV and Anti-Mnk levels

Assumption Checks

Test for Normality of residuals

Test	Statistics	p
Kolmogorov-Smirnov	0.0373	0.327
Shapiro-Wilk	0.9930	0.004

Q-Q Plot

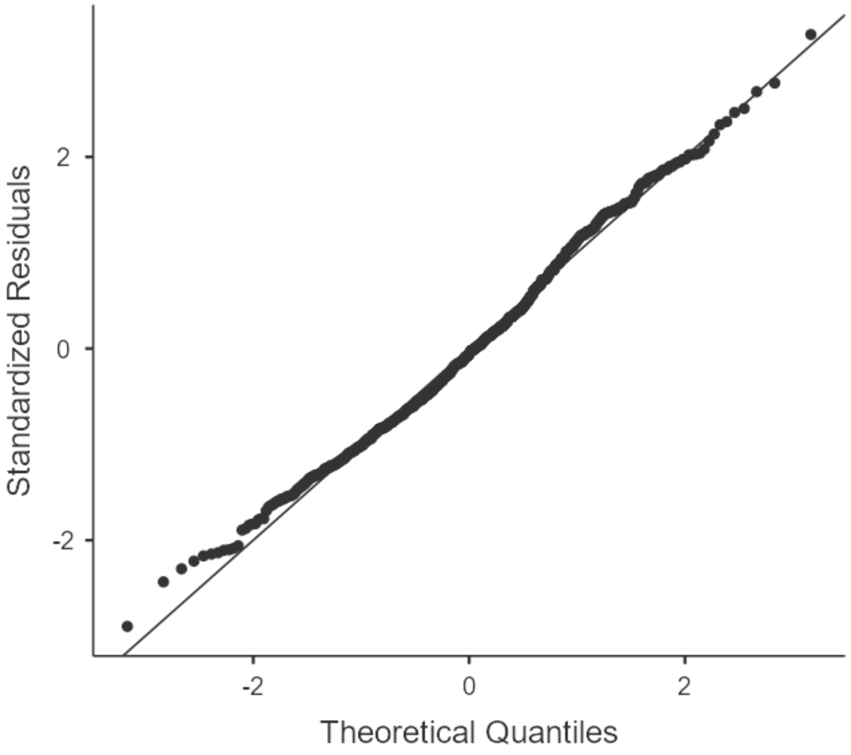

Residual histogram

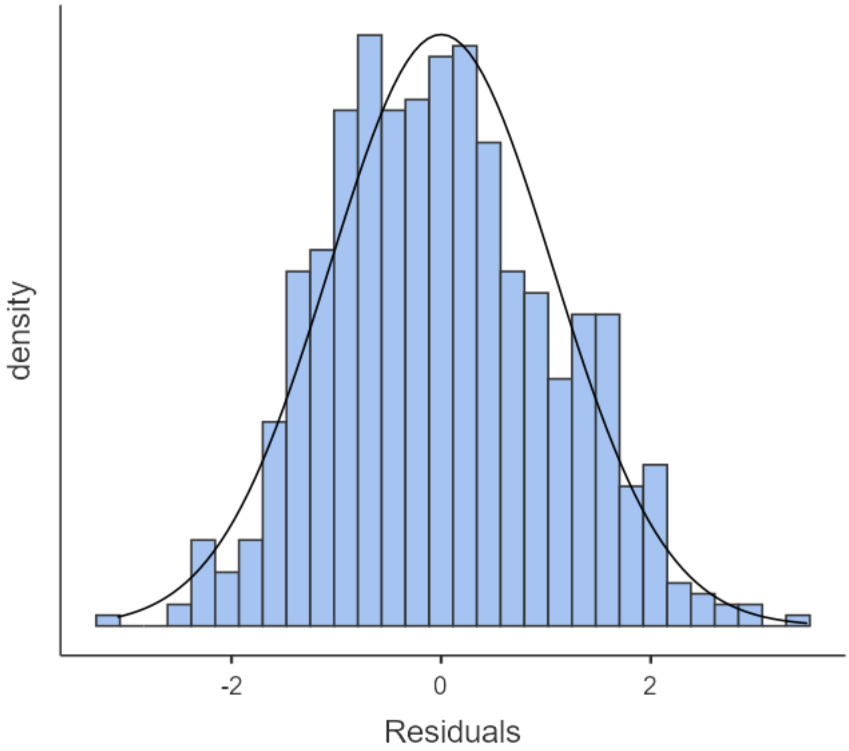

Assumption Checks

Test for Normality of residuals

Test	Statistics	p
Kolmogorov-Smirnov	0.128	0.838
Shapiro-Wilk	0.975	0.846

Q-Q Plot

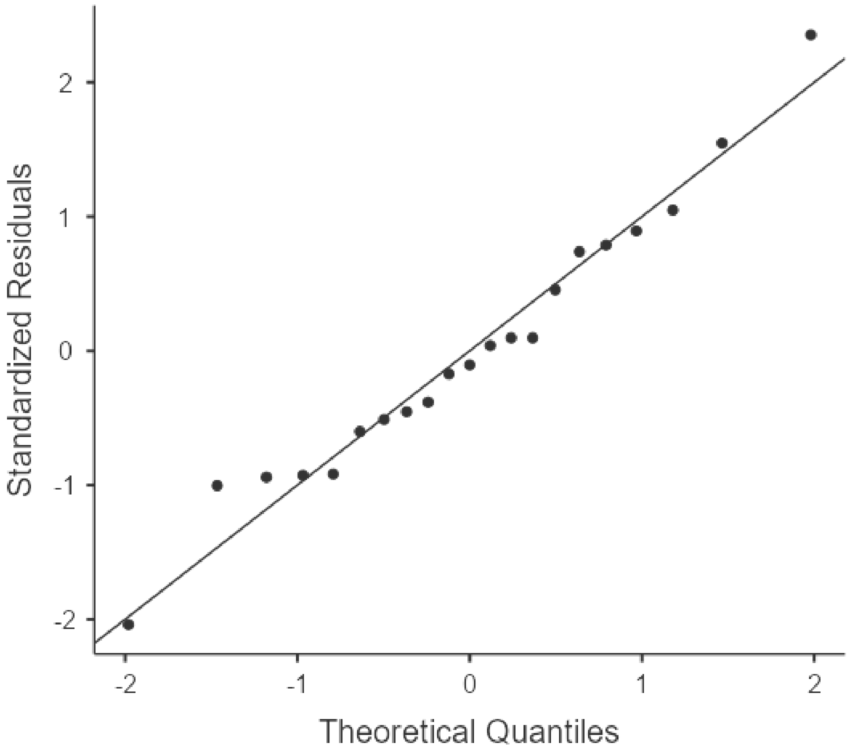

Residual histogram

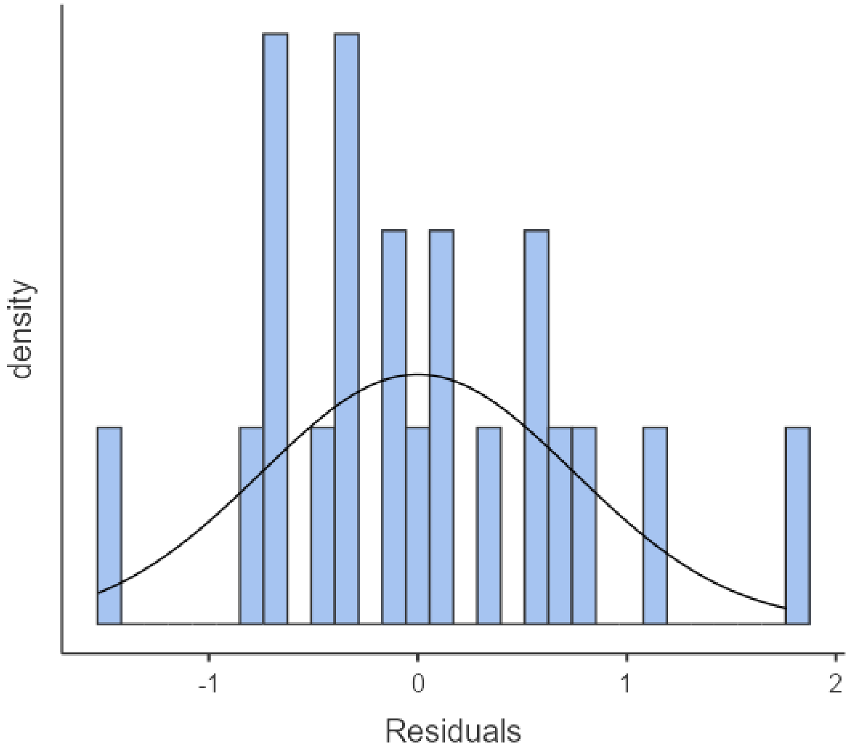

Relationship between Anti-Mnk levels and facial colouration

Assumption Checks

Test for Normality of residuals

Test	Statistics	p
Kolmogorov-Smirnov	0.149	0.689
Shapiro-Wilk	0.911	0.057

Q-Q Plot

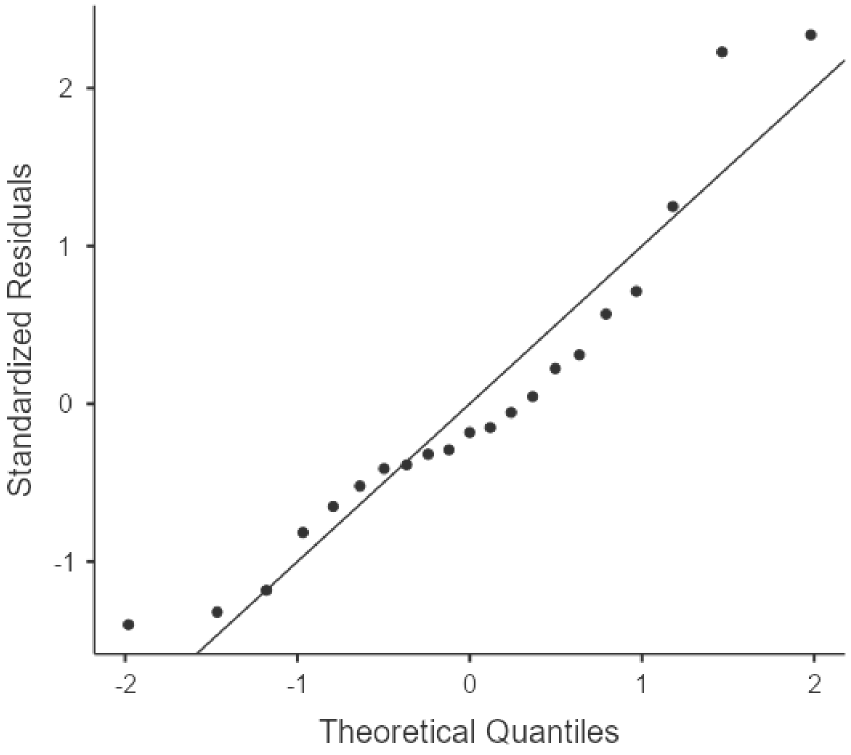

Residual histogram

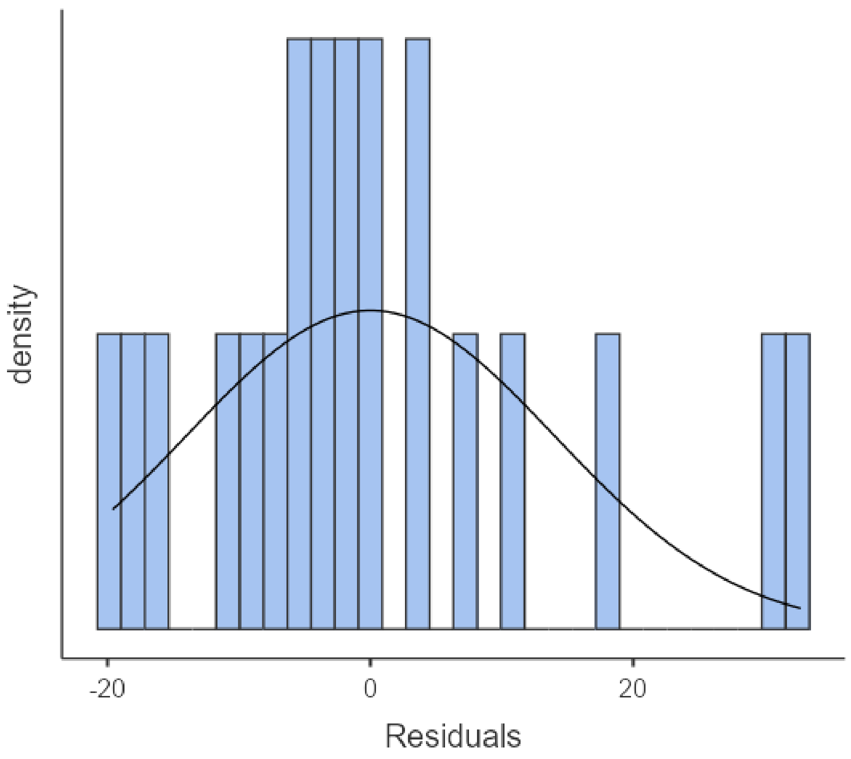

Assumption Checks

Test for Normality of residuals

Test	Statistics	p
Kolmogorov-Smirnov	0.0401	0.104
Shapiro-Wilk	0.9931	< .001

Q-Q Plot

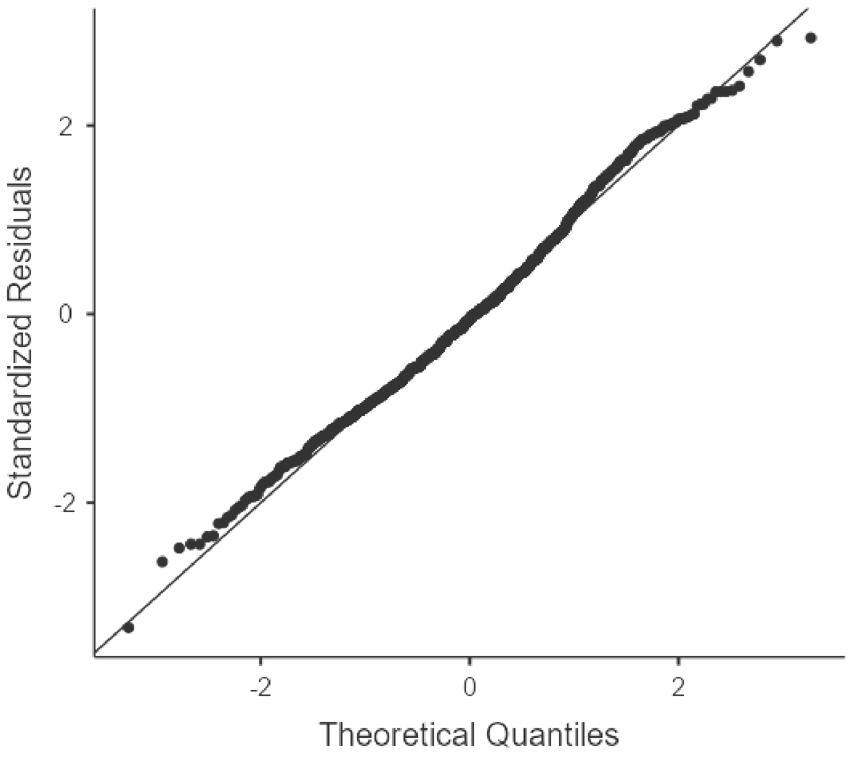

Residual histogram

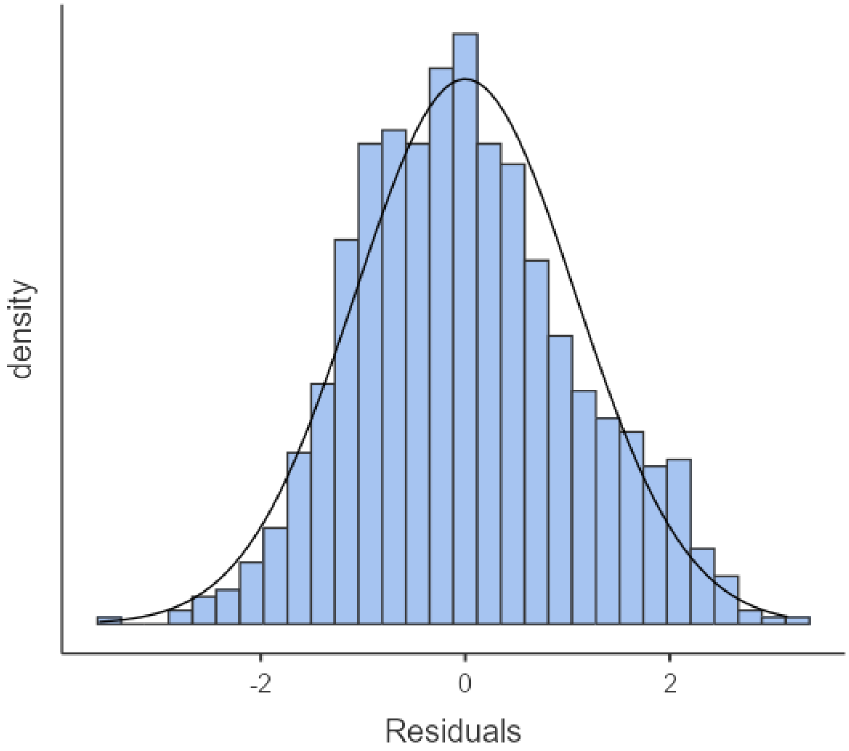

Assumption Checks

Test for Normality of residuals

Test	Statistics	p
Kolmogorov-Smirnov	0.0345	0.378
Shapiro-Wilk	0.9966	0.145

Q-Q Plot

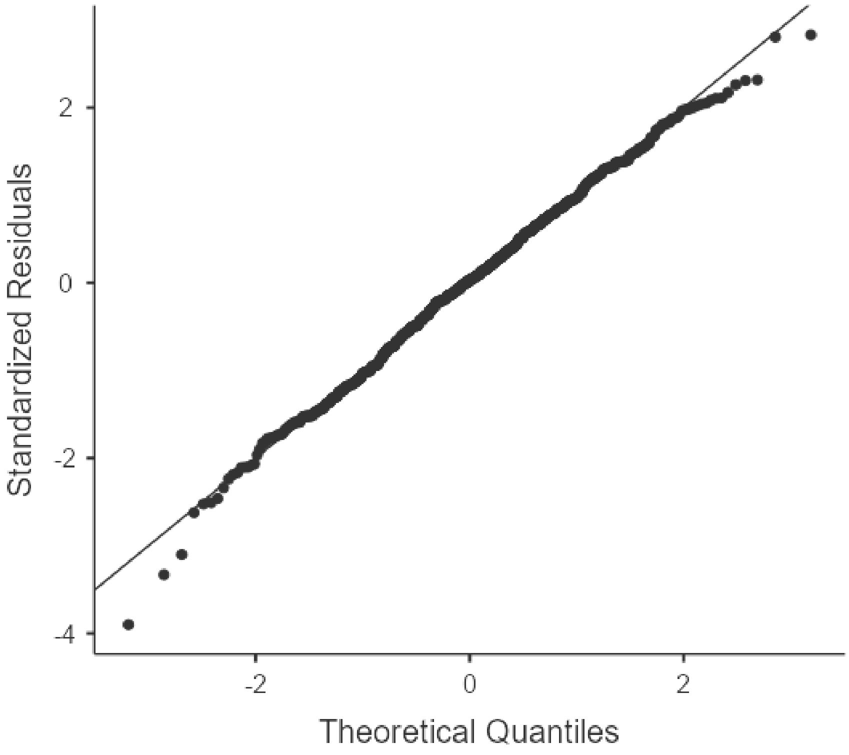

Residual histogram

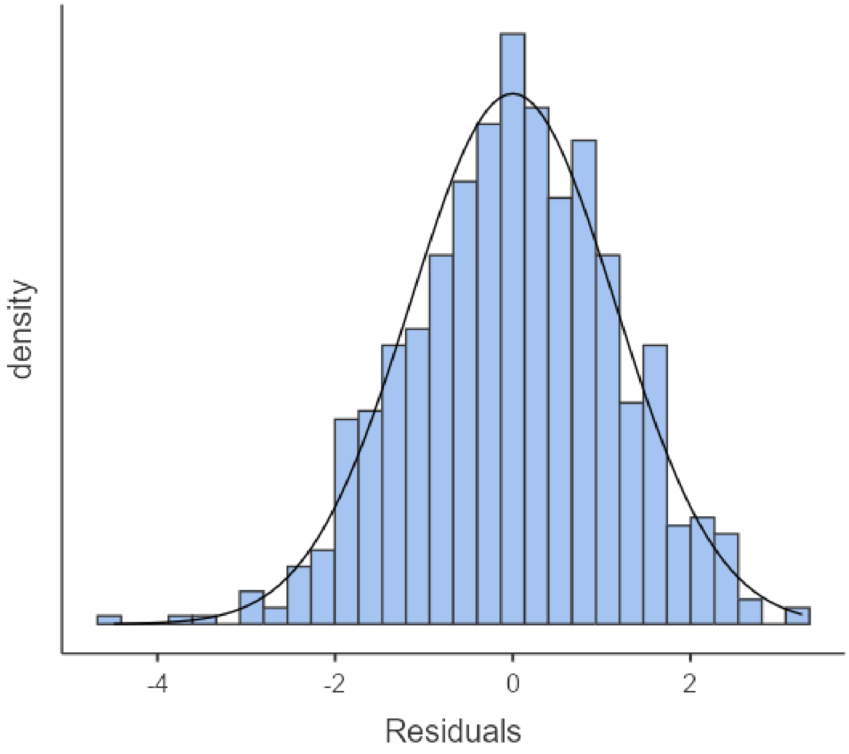

Assumption Checks

Test for Normality of residuals

Test	Statistics	p
Kolmogorov-Smirnov	0.203	0.310
Shapiro-Wilk	0.890	0.022

Q-Q Plot

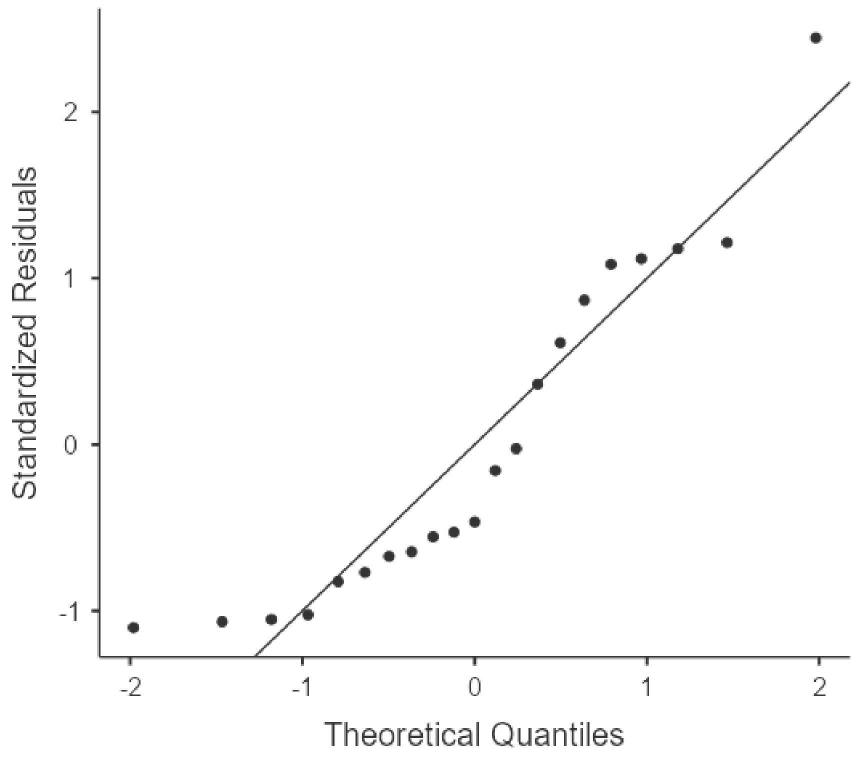

Residual histogram

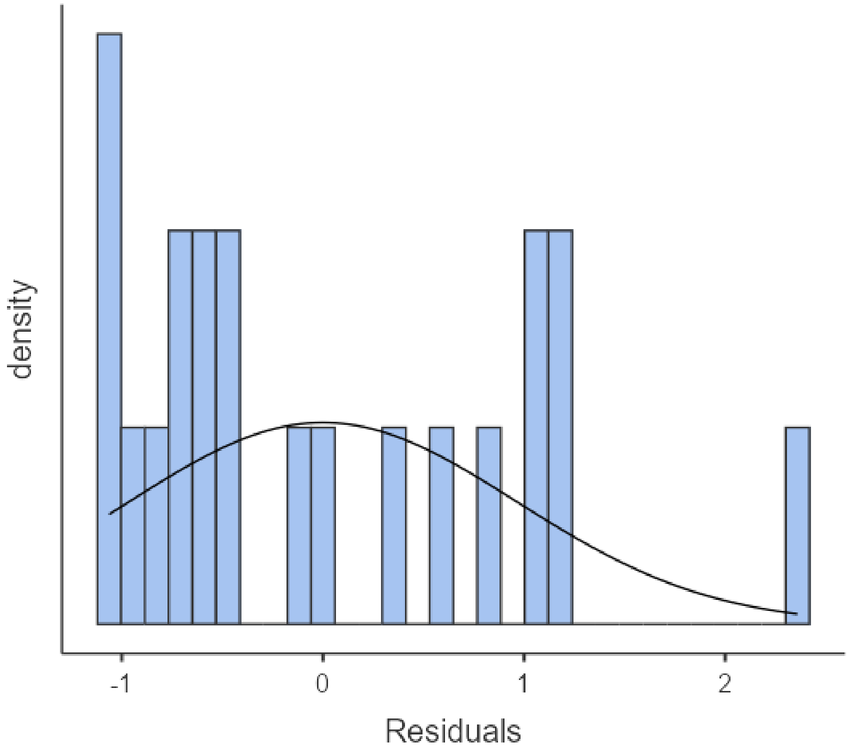

Relationship between Anti-Mnk levels, hormones and adipose tissue

Assumption Checks

Test for Normality of residuals

Test	Statistics	p
Kolmogorov-Smirnov	0.153	0.651
Shapiro-Wilk	0.899	0.034

Q-Q Plot

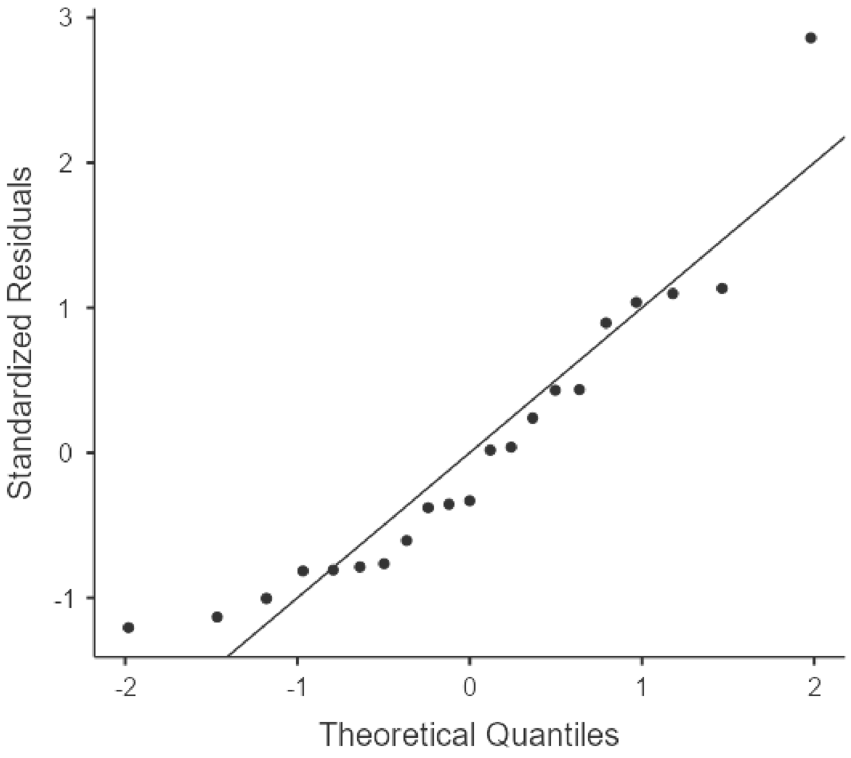

Residual histogram

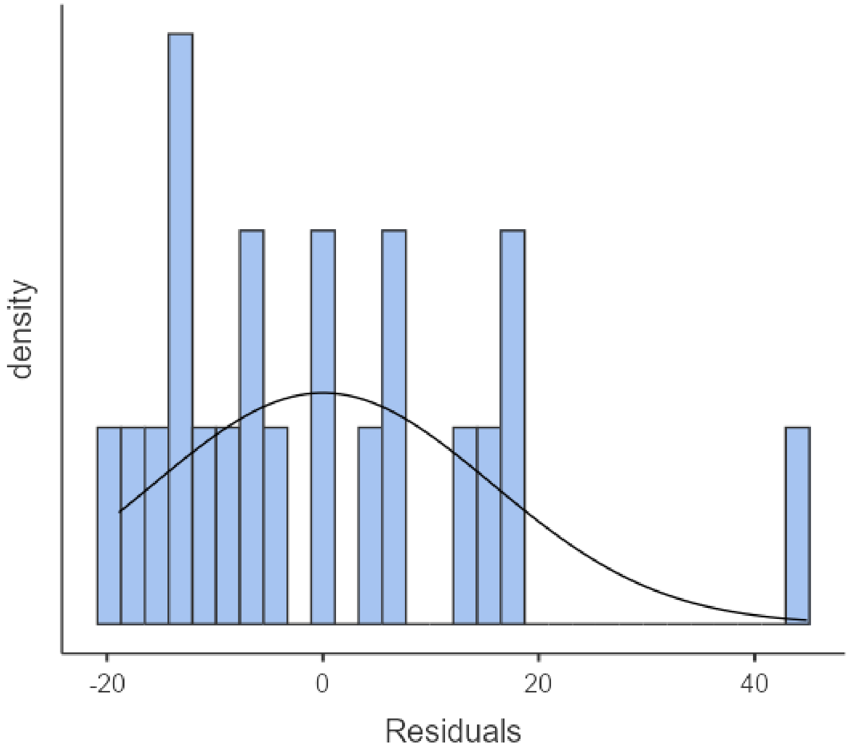

Relationship between perceived facial attractiveness and facial colouration

Assumption Checks

Test for Normality of residuals

Test	Statistics	p
Kolmogorov-Smirnov	0.0390	0.120
Shapiro-Wilk	0.9931	< .001

Q-Q Plot

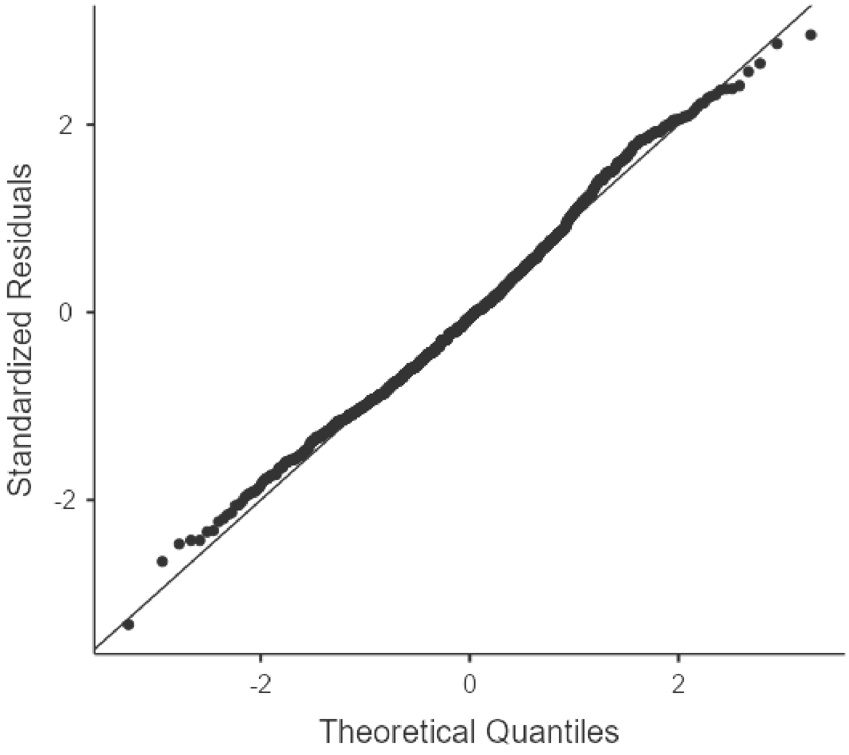

Residual histogram

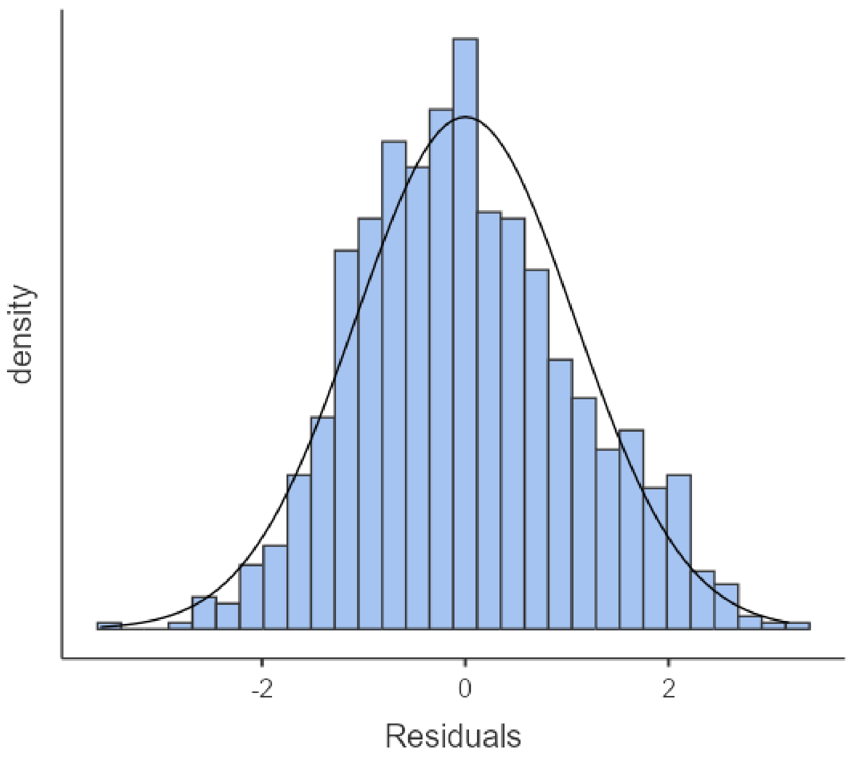

Assumption Checks

Test for Normality of residuals

Test	Statistics	p
Kolmogorov-Smirnov	0.0254	0.758
Shapiro-Wilk	0.9970	0.236

Q-Q Plot

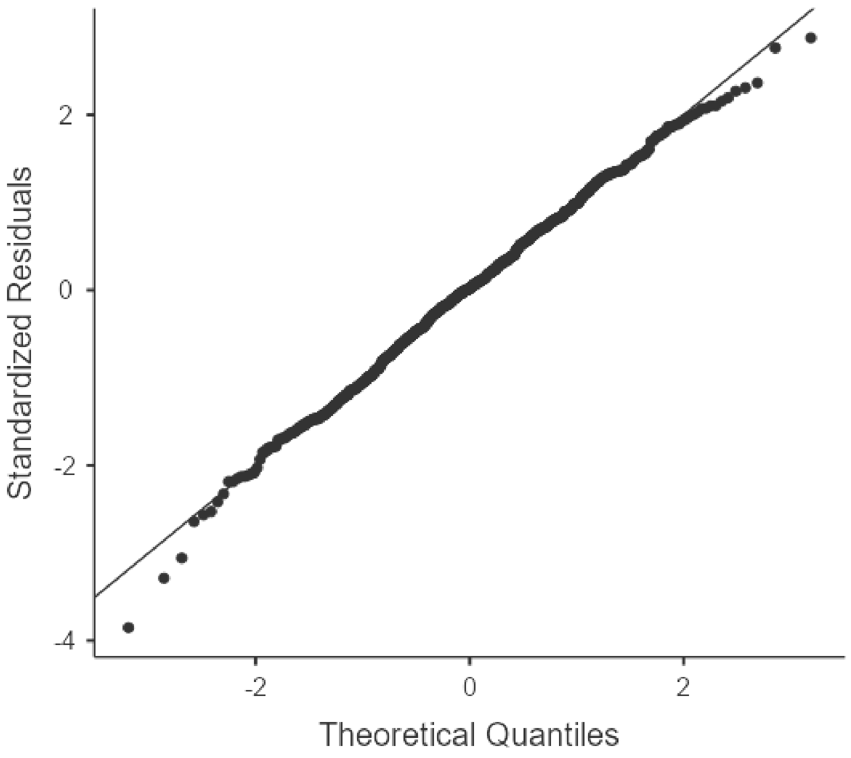

Residual histogram

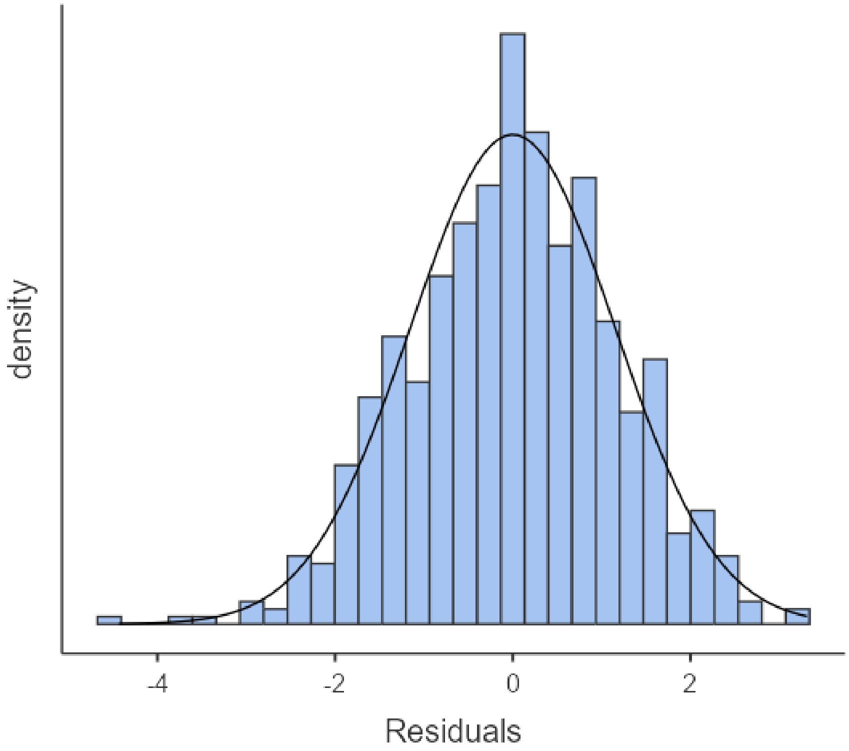

Relationship between perceived cheek patch healthiness and facial colouration

Assumption Checks

Test for Normality of residuals

Test	Statistics	p
Kolmogorov-Smirnov	0.0383	0.298
Shapiro-Wilk	0.9921	0.002

Q-Q Plot

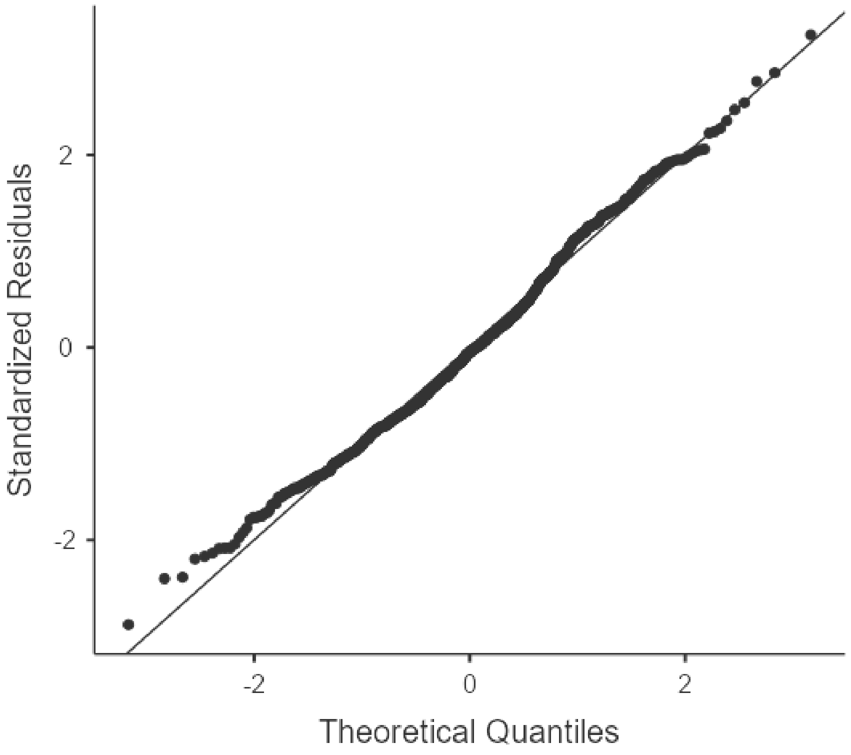

Residual histogram

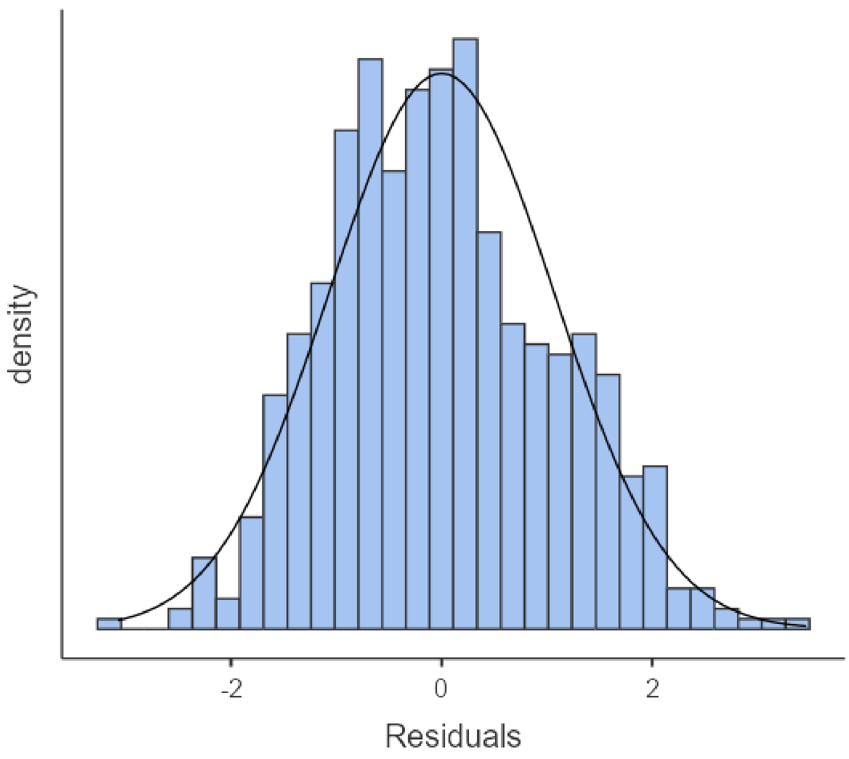

Supplement: Supplementary file 1 — Supplementary Information 1. [file 41598_2022_22866_MOESM1_ESM.pdf]
