# Supplementary material for: Attractive and healthy-looking male faces do not show higher immunoreactivity
Source: Sci Rep. 2022 Nov 1;12:18432. doi: 10.1038/s41598-022-22866-x (PMC9626598; doi:10.1038/s41598-022-22866-x)
Supplement: Supplementary file 3 — Supplementary Information 3. [file 41598_2022_22866_MOESM3_ESM.pdf]

Observed power for individual fixed effects using SimR R package

Model	Dependent variable	Fixed effect	Observed power (%) *		
			Mean	95% CI LL	95% CI UL
attractiveness rating ~ Anti_HAV_3_Anti_HAV_1 + Anti_Mnk_3_Anti_Mnk_1 + (1|rater) + (1|target)	Perceived facial attractiveness ~	AntiHav	20	16.95	24.2
		AntiMnk	9	6.64	11.86
healthiness rating ~ Anti_HAV_3_Anti_HAV_1 + Anti_Mnk_3_Anti_Mnk_1 + (1|rater) + (1|target)	Perceived facial healthiness ~	AntiHav	< 1	0	0.07
		AntiMnk	17.2	13.99	20.78
patch rating ~ Anti_HAV_3_Anti_HAV_1 + Anti_Mnk_3_Anti_Mnk_1 + (1|rater) + (1|target)	Perceived patch healthiness ~	AntiHav	< 1	0	0.07
		AntiMnk	24.4	20.7	28.41
attractiveness rating ~ testosteron_1 + kortizol_1 + Adipose + (1|rater) + (1|target)	Perceived facial attractiveness ~	Testosterone	0.64	0.44	0.89
		Cortisol	34.6	30.4	38.95
		Adiposity	55.4	50.92	59.81
healthiness rating ~ testosteron_1 + kortizol_1 + Adipose + (1|rater) + (1|target)	Perceived facial healthiness ~	Testosterone	17.6	14.36	21.23
		Cortisol	60.4	55.96	64.71
		Adiposity	11.6	8.93	14.74

Note: * 95%CI estimated with nsim = 500

Below you can find Power curve plots generated for each fixed effect with data simulation using the SimR package. We chose to extend the original sample (N = 21) to 100 with 500 simulations (nsim). R script can be found among other supplementary materials of the manuscript. The green vertical line in each plot represents the original sample size (N=21).

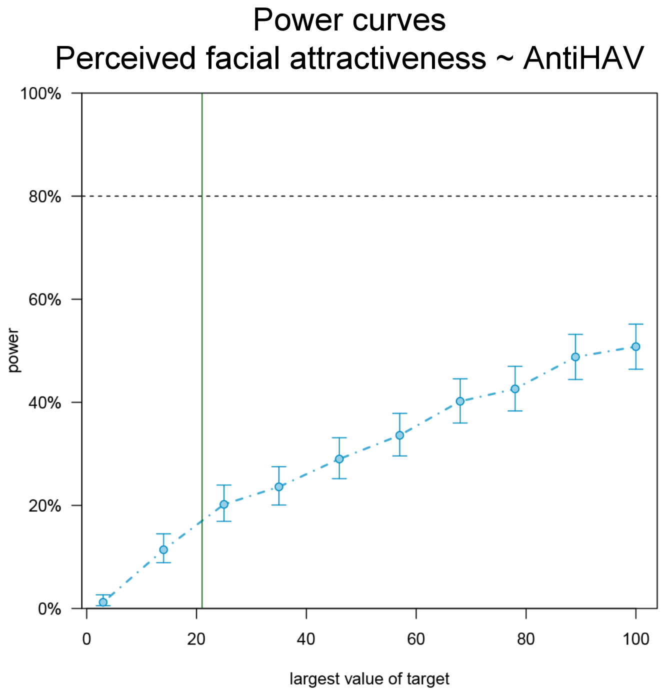
Power curves
Perceived facial attractiveness ~ AntiHAV
power
largest value of target
0%
20%
40%
60%
80%
100%
0
20
40
60
80
100

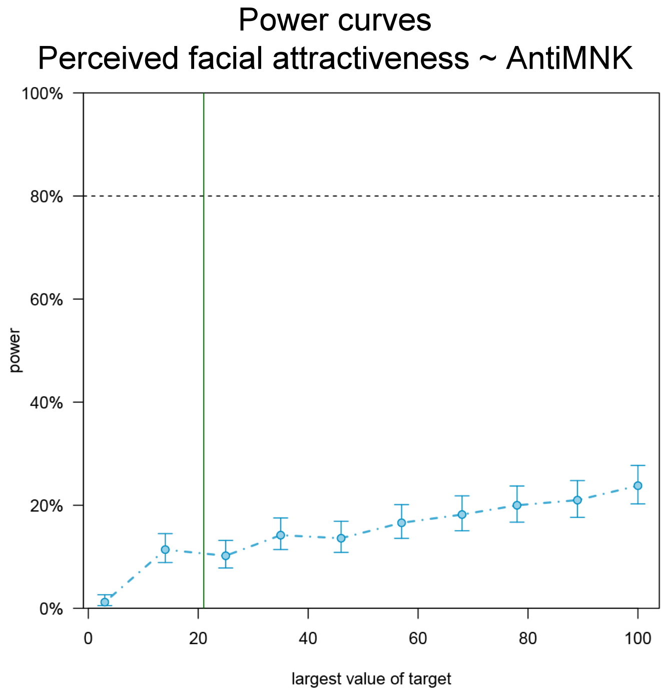
Power curves
Perceived facial attractiveness ~ AntiMNK
power
largest value of target
0%
20%
40%
60%
80%
100%
0
20
40
60
80
100

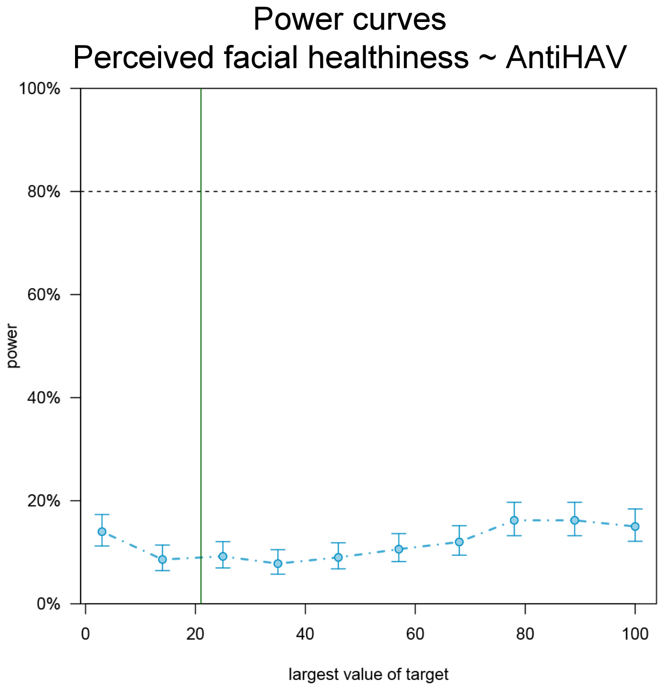
Power curves
Perceived facial healthiness ~ AntiHAV
power
largest value of target
0%
20%
40%
60%
80%
100%
0
20
40
60
80
100

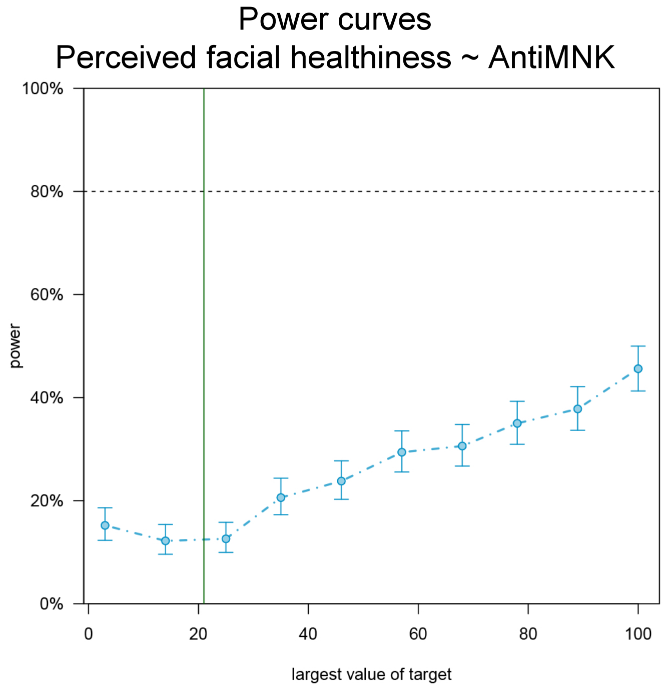
Power curves
Perceived facial healthiness ~ AntiMNK
power
largest value of target
0%
20%
40%
60%
80%
100%
0
20
40
60
80
100

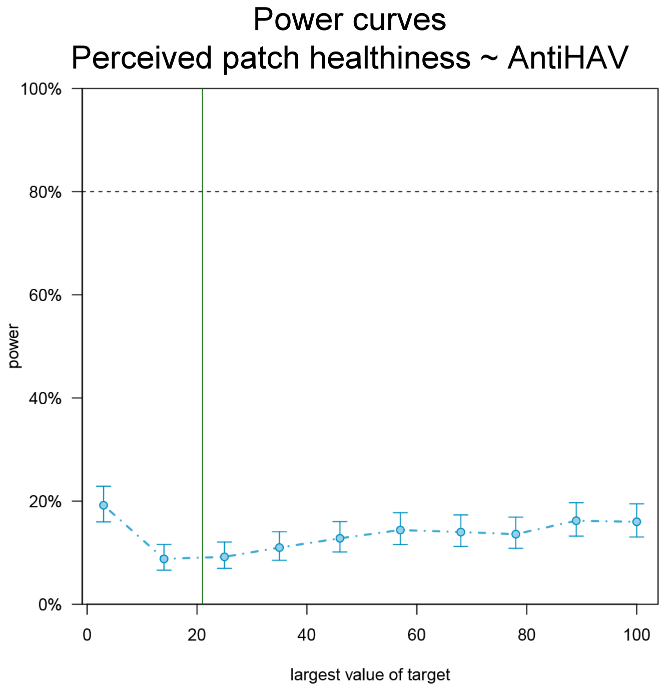
Power curves
Perceived patch healthiness ~ AntiHAV
power
largest value of target
0%
20%
40%
60%
80%
100%
0
20
40
60
80
100

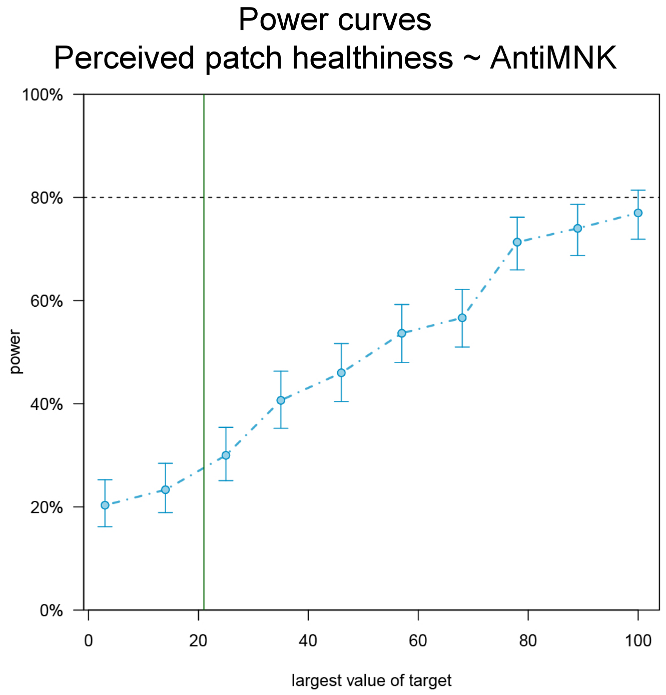
Power curves
Perceived patch healthiness ~ AntiMNK
power
largest value of target
0%
20%
40%
60%
80%
100%
0
20
40
60
80
100

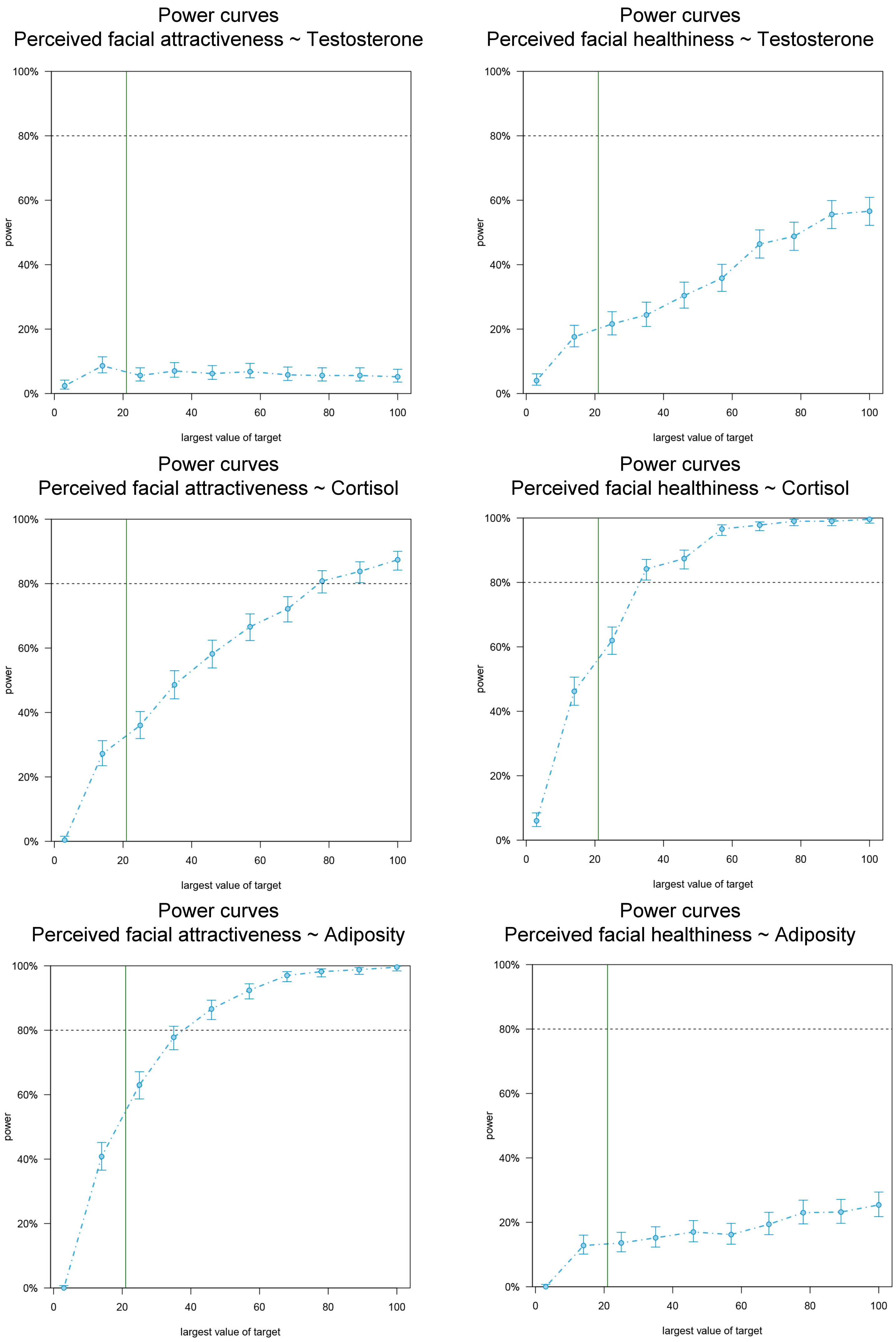
Power curves
Perceived facial attractiveness ~ Testosterone
Power curves
Perceived facial healthiness ~ Testosterone
Power curves
Perceived facial attractiveness ~ Cortisol
Power curves
Perceived facial healthiness ~ Cortisol
Power curves
Perceived facial attractiveness ~ Adiposity
Power curves
Perceived facial healthiness ~ Adiposity
power
0% 20% 40% 60% 80% 100%
0 20 40 60 80 100
largest value of target
